# *Bank1* modulates the differentiation and molecular profile of key B cell populations in autoimmunity

**DOI:** 10.1172/jci.insight.179417

**Published:** 2024-08-20

**Authors:** Gonzalo Gómez Hernández, Toro Domínguez, Georgina Galicia, María Morell, Marta E. Alarcón-Riquelme

**Affiliations:** 1 Department of Functional Genomics, GENyO, Center for Genomics and Oncological Research Pfizer, University of Granada, Andalusian Regional Government, Parque Tecnológico de la Salud, Granada, Spain.; 2 Department of Physiology, Faculty of Pharmacy, University of Granada, Campus de Cartuja, Granada, Spain.; 3 Institute for Environmental Medicine, Karolinska Institute, Stockholm, Sweden.

**Keywords:** Autoimmunity, Genetics, Adaptive immunity, Autoimmune diseases, Mouse models

## Abstract

This study aimed at defining the role of the B cell adaptor protein BANK1 in the appearance of age-associated B cells (ABCs) in 2 SLE mouse models (TLR7.tg6 and imiquimod-induced mice), crossed with *Bank1^–/–^* mice. The absence of *Bank1* led to a significant reduction in ABC levels, also affecting other B cell populations. To gain deeper insights into their differentiation pathway and the effect of *Bank1* on B cell populations, a single-cell transcriptome assay was performed. In the TLR7.tg6 model, we identified 10 clusters within B cells, including an ABC-specific cluster that was decreased in *Bank1*-deficient mice. In its absence, ABCs exhibited an antiinflammatory gene expression profile, while being proinflammatory in *Bank1*-sufficient lupus-prone mice. Trajectory analyses revealed that ABCs originated from marginal zone and memory-like B cells, ultimately acquiring transcriptional characteristics associated with atypical memory cells and long-lived plasma cells. Also, *Bank1* deficiency normalized the presence of naive B cells, which were nearly absent in lupus-prone mice. Interestingly, *Bank1* deficiency significantly reduced a distinct cluster containing IFN-responsive genes. These findings underscore the critical role of *Bank1* in ABC development, affecting early B cell stages toward ABC differentiation, and the presence of IFN-stimulated gene–containing B cells, both populations determinant for autoimmunity.

## Abstract 2

*Bank1* has a major role in the development and transcriptomic pattern of both age-associated B cells and IFN-responsive B cells in autoimmunity.

## Introduction

Systemic lupus erythematosus (SLE) is a multiorganic autoimmune disease of unknown etiology, caused by the complex combination of genetic and environmental factors (1). The disease is characterized by the loss of tolerance and activation of the immune response, leading to an excessive production of autoantibodies against various cellular components (2). These autoantibodies, primarily to nucleic acids or RNPs, such as Smith antigens, form immunocomplexes (ICs), which are deposited in various tissues with resultant inflammation, the production of proinflammatory cytokines, and damage (2, 3). The hyperactivation and dysregulation of B cell function is a hallmark of the disease (4). In addition to secreting pathogenic autoantibodies and cytokines, B cells are also antigen-presenting cells (APCs) (5). Hence, a deeper comprehension of the development and differentiation of various populations of B cells and their contribution to autoimmunity is essential for a better understanding of the disease.

The importance of the B cell scaffold with ankyrin repeats 1 (*BANK1*) in autoimmunity stems from a genome-wide association study that identified variants within this gene as inferring susceptibility for human SLE (6). BANK1, first identified by Yokoyama et al. (7), is an adaptor protein primarily expressed by B cells and, to a lesser extent, by myeloid and plasmacytoid DCs (7, 8). The *BANK1* gene is located on human chromosome 4 (4q24, syntenic with mouse chromosome 3), and its major isoform (full-length) has 755 amino acids. An alternative spliced isoform lacking exon 2 (Δ2) encodes a protein without a TLR-binding domain (TIR domain) (6, 9). Decreased expression of this isoform and increased expression of the full-length isoform have been linked, genetically, to risk for SLE (6, 10).

BANK1 protein is involved in different B cell signaling pathways (11). BANK1 becomes tyrosine phosphorylated and binds the Src family kinases LYN and BLK upon BCR stimulation (7, 12). BANK1 also interacts with PLC-γ2 through several tyrosine domains; this interaction is modulated by BLK (13). BANK1 attenuates CD40-mediated AKT activation (8). Following the PI3K/AKT pathway, the expression of the transcription factors FOXO1 and AICDA appear to increase in patients with human *BANK1* genetic risk variants, resulting in an increase in plasma cells, memory B cells (MBCs), and germinal center B cells (14). But among all these pathways, the role of BANK1 in the endosomal TLR7 signaling appears to be highly relevant for SLE. BANK1 interacts with the adaptor molecule myeloid differentiation primary response gene 88 (MyD88) through the interaction with its TIR domain, and with the TNF receptor–associated factor 6 (TRAF6) through TRAF6-specific binding sites located in the BANK1 protein, triggering the production of several proinflammatory cytokines (10).

To date, the exact role of BANK1 in inflammatory diseases and autoimmunity remains incompletely understood. Experiments in *Bank1*-deficient mice reported a reduction in the production of the proinflammatory cytokine IL-6 after TLR9 stimulation (15) by CpG induction and was linked to the MAPK p38 phosphorylation. *Bank1* deficiency affected the MNK1/2/eIF4E/eIF4G pathway of the translation initiation process controlled by p38 by reducing CpG-induced MNK1/2 and eIF4E phosphorylation (16). Following the report of the genetic association of *BANK1* with SLE (6), the same group (17) showed that *Bank1* deficiency reduced major lupus phenotypes in B6.*Sle1*.*yaa* lupus-prone mice, including the production of total IgG, IgG2c, and IgG anti-dsDNA autoantibodies, as well as serum levels of IL-6 and BAFF. *Bank1* deficiency also restored the cellular phenotypes of splenic lymphocytes and myeloid cells. In vitro experiments stimulating through TLR7 and TLR8 showed that *Bank1* regulates TLR7-induced signaling pathways in B cells, leading to the reduced expression of *Aicda*, *Irf1* and *Irf9*, *Stat1*, and type I IFN-β in B6.*Sle1*.*yaa*.*Bank1^–/–^* mice (17).

Recent studies have identified a distinct mature B cell subset present in a small percentage of normal mice, known as age-associated B cells (ABCs), which present a unique phenotype and accumulate with age (18, 19). Different markers have been used to identify this population. Hao et al. described ABCs as CD19^+^ splenic B cells lacking CD21, CD23, CD93, and CD43 (18), whereas Rubtsov et al. defined them as the double-positive expression of CD11b and CD11c on CD19^+^ B cells (19).

ABCs have been implicated in autoimmunity in both mice and humans. This population is thought to be driven by TLR7 signaling, as the cells proliferate robustly upon stimulation and activation of this endosomal receptor (19, 20) and secrete autoantibodies (21, 22). A first study showed that, in NZB/WF1 and *Mer^–/–^* SLE mouse models, ABCs were significantly expanded with age and secreted anti–chromatin IgG autoantibodies in vitro (19). In addition to the surface phenotype, it has been recently reported that most ABCs express the transcription factor T-bet (22, 23), encoded by the *Tbx21* gene. T-bet promotes IgG isotype class-switch recombination to IgG2a/c in B cells, and it is also involved in pathogenic autoantibody production (24). Subsequent studies in mouse models revealed that CD11c^+^T-bet^+^ ABCs are required for anti–chromatin antibodies in the bm12 cGVHD SLE model (22). Using the SWEF protein (SWAP-70 and DEF6) double-KO lupus model, Manni et al. demonstrated that differentiation to T-bet^+^ ABCs was IL-21 dependent and controlled by *Irf5* (25). Several studies have shown that T-bet deficiency prevents ABC differentiation and autoantibody production in mice, suggesting that T-bet expression is required for ABC formation (21, 25).

In humans, there is an ABC-like population with similar phenotypic and functional features termed double-negative 2 (DN2), lacking IgD and CD27, observed in patients with SLE (21, 26). An increase in circulating ABCs in peripheral blood was strongly correlated with disease activity, and these cells were highly enriched in autoantibody-producing cells of various specificities (27).

Given the significance of *Bank1* in the TLR7 pathway and the influence of this signaling pathway on the ABC population, the present study aimed to define the role of *Bank1* in the appearance of ABCs in *Tlr7*-dependent SLE mouse models. Using, among others, splenocyte single-cell transcriptome analysis, and 2 lupus models crossed with the KO for *Bank1*, a transgenic model of *Tlr7* (TLR7.tg6) (28), and a TLR7-pathway imiquimod-induced (IMQ-induced) disease model (29), we show that *Bank1* has a fundamental role in the differentiation of key B cell populations in autoimmunity and B cell functions dependent on TLR7 signaling.

## Results

### Bank1 deficiency decreases autoantibody production in TLR7.tg6 and IMQ-treated lupus-prone mice.

The effects of *Bank1* deficiency on various lupus phenotypes in the TLR7.tg6 model, by 32 weeks of age, and the IMQ-induced model, by 20 weeks of age, were analyzed. *Bank1* deficiency slightly improved the survival rate of the TLR7.tg6 model (85.7%–100%) and the IMQ-induced model (76.9%–90%), but differences were not significant (Supplemental Figure 1A; supplemental material available online with this article; https://doi.org/10.1172/jci.insight.179417DS1).

**Figure 1:**
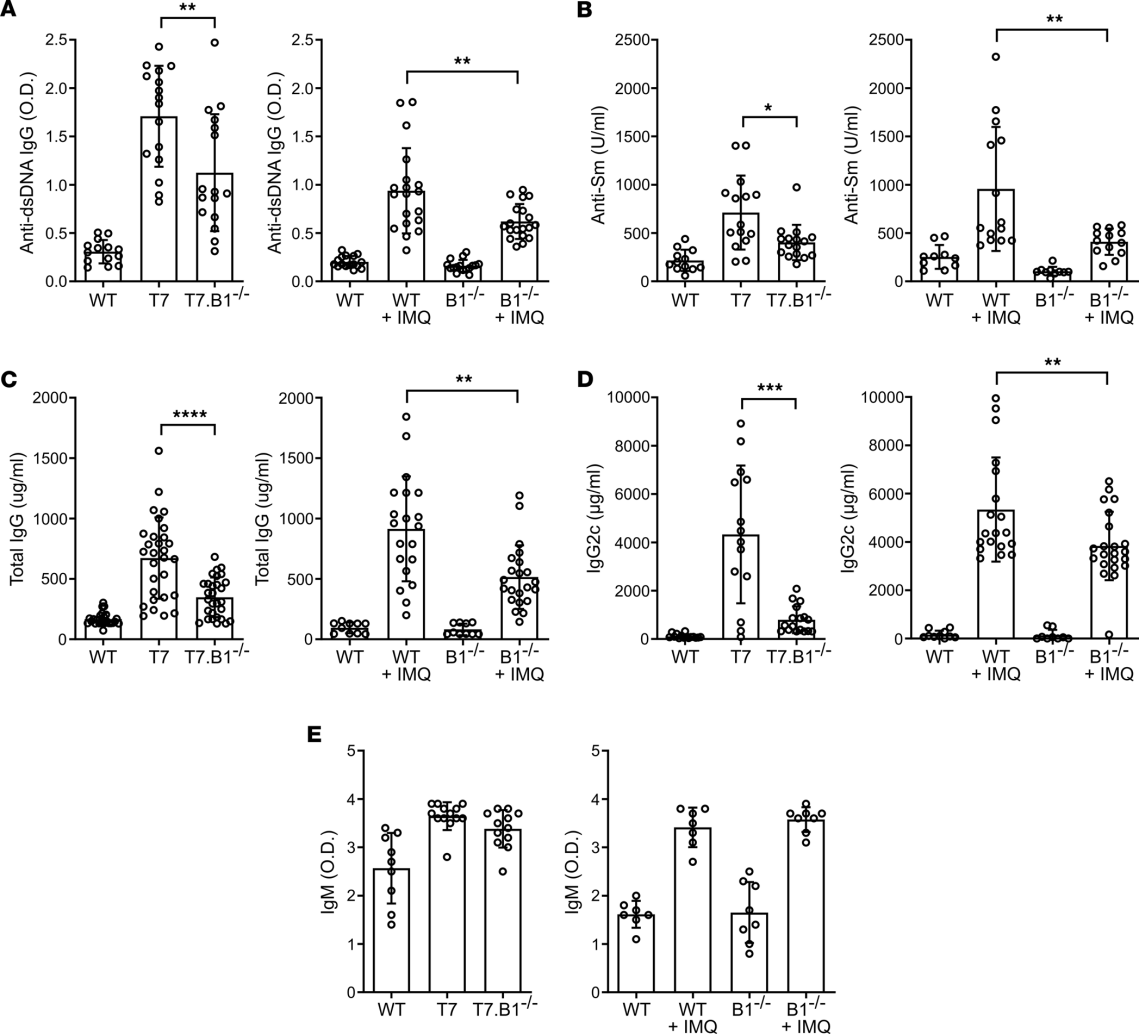
*Bank1* deficiency improves major lupus phenotypes in TLR7.tg6 and in IMQ-treated mice. Serum levels of antibodies were quantified by ELISA in the TLR7.tg6 model (32 weeks of age), and in the IMQ-induced model (20 weeks of age). (**A**) Serum levels of IgG antidsDNA antibodies in optical density (OD) at 1:1,000 dilution. Total mice analyzed: WT (*n* = 14), T7 (*n* = 16), T7.B1^–/–^ (*n* = 16); and WT (*n* = 16), WT + IMQ (*n* = 19), B1^–/–^ (*n* = 16), B1^–/–^ + IMQ (*n* = 19). (**B**) Serum levels of anti-Sm antibodies (U/mL) at 1:100 dilutions. Total mice analyzed: WT (*n* = 12), T7 (*n* = 15), T7.B1^–/–^ (*n* = 16); and WT (*n* = 10), WT + IMQ (*n* = 14), B1^–/–^ (*n* = 10), B1^–/–^ + IMQ (*n* = 13). (**C**) Serum levels of total IgG (μg/mL) at 1:100,000 dilutions. Total mice analyzed: WT (*n* = 25), T7 (*n* = 29), T7.B1^–/–^ (*n* = 29); and WT (*n* = 10), WT + IMQ (*n* = 20), B1^–/–^ (*n* = 10), B1^–/–^ + IMQ (*n* = 22). (**D**) Serum levels of IgG2c (μg/mL) at 1:10,000 dilution. Total mice analyzed: WT (*n* = 15), T7 (*n* = 15), T7.B1^–/–^ (*n* = 17); and WT (*n* = 10), WT + IMQ (*n* = 19), B1^–/–^ (*n* = 9), B1^–/–^ + IMQ (*n* = 22). (**E**) Serum levels of IgM in optical density (OD) at 1:5,000 dilution. Total mice analyzed: WT (*n* = 9), T7 (*n* = 13), T7.B1^–/–^ (*n* = 12); and WT (*n* = 7), WT + IMQ (*n* = 7), B1^–/–^ (*n* = 8), B1^–/–^ + IMQ (*n* = 8). Each point represents 1 individual mouse. Data are shown as mean ± SEM. Mann-Whitney *U* test with Welch’s correction was used to test statistical significance.

Serum levels of IgG anti-dsDNA antibodies were strongly increased in TLR7.tg6 and IMQ-treated WT mice compared with WT or untreated mice, respectively, and were significantly reduced in TLR7.tg6.*Bank1^–/–^* or IMQ-treated *Bank1^–/–^* mice (Figure 1A). A similar pattern was observed with anti-Sm antibodies in both models (Figure 1B). Total IgG levels (Figure 1C) and the IgG2c isotype (Figure 1D) were also increased in the serum of TLR7.tg6 and IMQ-treated WT mice as compared with WT mice or untreated mice, respectively. In the absence of *Bank1*, they were significantly reduced. Besides, *Bank1* deficiency reduced serum levels of IgG1 compared with TLR7.tg6 mice, but differences were not significant in the IMQ-induced model (Supplemental Figure 1B). Also in accordance to previous results (17), total IgM levels were not modified by *Bank1* deficiency (Figure 1E). These results suggest that *Bank1* deficiency limits major lupus phenotypes by reducing the pathogenic autoantibody production in SLE murine models.

### Bank1 deficiency restores the cellular phenotypes of splenic B-lymphocyte populations TLR7.tg6 and IMQ-treated lupus-prone mice.

**Figure 2:**
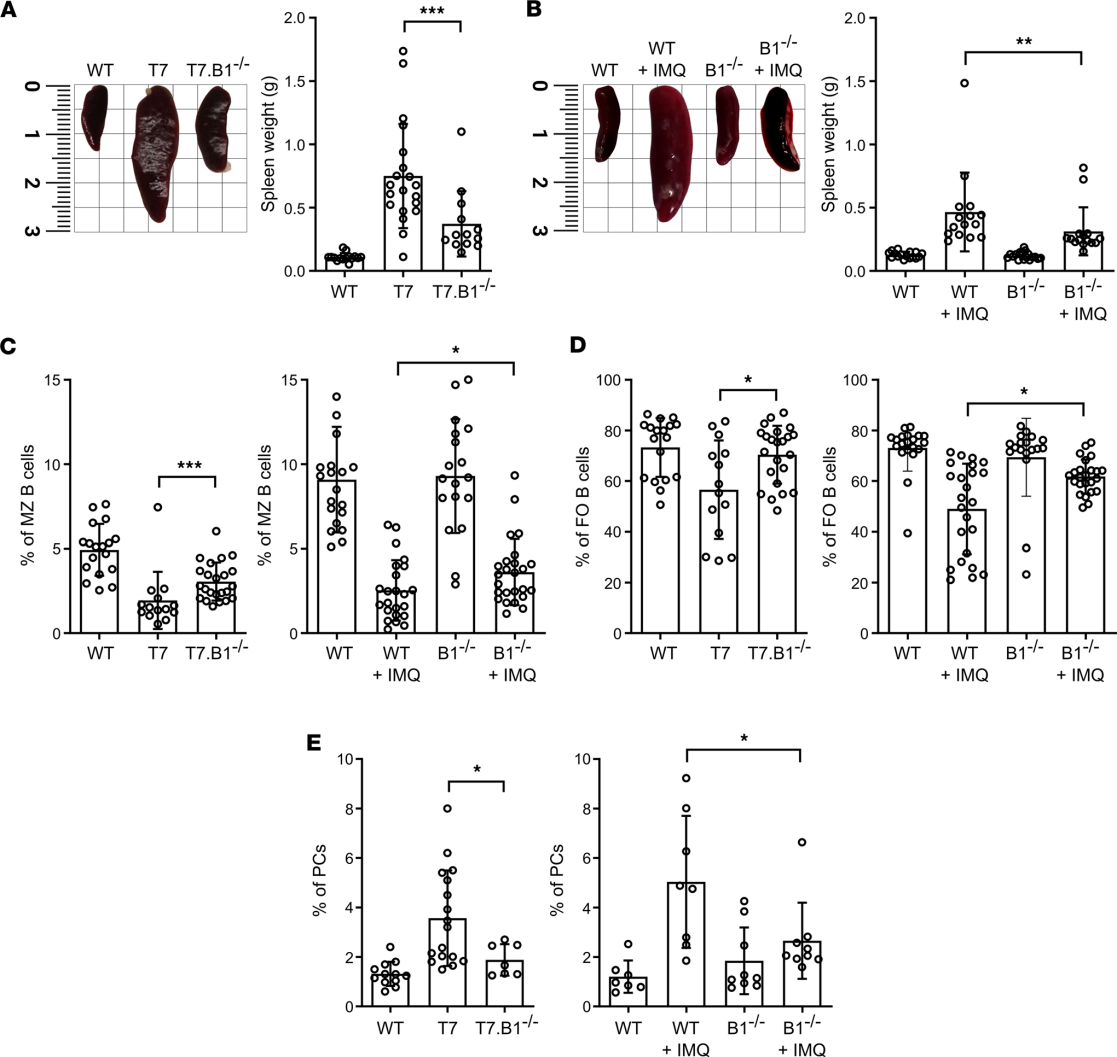
*Bank1* deficiency reduces splenomegaly and restores main splenic B lymphocyte populations from TLR7.tg6 and IMQ-treated mice. (**A**) Representative image of the size of the spleen from the TLR7.tg6 model, by 32 weeks of ages (on the left). The spleen weight from each group (on the right). Total mice analyzed: WT (*n* = 15), T7 (*n* = 20), T7.B1^–/–^ (*n* = 13). (**B**) Representative image of the size of the spleen from the IMQ-induced model, by 20 weeks of age (on the left). The spleen weight from each group (on the right). Total mice analyzed: WT (*n* = 18), WT + IMQ (*n* = 18), B1^–/–^ (*n* = 22), B1^/–^ + IMQ (*n* = 19). (**C** and **D**) Frequency of MZ B cells as CD21^+^CD23^–^ and FO B cells as CD21^–^ CD23^+^ among CD19^+^ B cells from the spleens of TLR7.tg6 and IMQ-induced models. Total mice analyzed: WT (*n* = 18), T7 (*n* = 14), T7.B1^–/–^ (*n* = 23); and WT (*n* = 20), WT + IMQ (*n* = 24), B1^/–^ (*n* = 18), B1^/–^ + IMQ (*n* = 25). (**E**) Frequency of PCs as CD138^+^ from the spleens of TLR7.tg6 and IMQ-induced models. Total mice analyzed: WT (*n* = 12), T7 (*n* = 17), T7.B1^–/–^ (*n* = 7); and WT (*n* = 7), WT + IMQ (*n* = 8), B1^–/–^ (*n* = 9), B1^–/–^ + IMQ (*n* = 9). Each point represents 1 individual mouse. Data are shown as mean ± SEM. Mann-Whitney *U* test with Welch’s correction was used to test statistical significance.

The spleen size in the TLR7.tg6 group and in the IMQ-induced model was significantly larger than in *Bank1^–/–^* mice, although not reaching sizes of healthy control and untreated mice (Figure 2, A and B).

We examined by flow cytometry the extent to which *Bank1* deficiency restored to normal some B cell phenotypes modified by the autoimmune process. *Bank1* deficiency significantly increased the frequency of marginal zone (MZ) B cells (CD19^+^CD21^+^CD23) and follicular (FO) B cells (CD19^+^CD21^–^CD23^+^) in TLR7.tg6 mice (Figure 2C) and in IMQ-treated mice (Figure 2D). Also, the increased percentage of CD138^+^ plasma cells (PCs) in both lupus models, compared with healthy control and untreated mice, was reduced in *Bank1^–/–^* mice (Figure 2E). These findings show the effect of *Bank1* deficiency on TLR7-mediated splenomegaly and main B cell subsets, during autoimmunity.

### ABCs are reduced in the absence of Bank1 in TLR7.tg6 and IMQ-treated lupus-prone mice.

**Figure 3:**
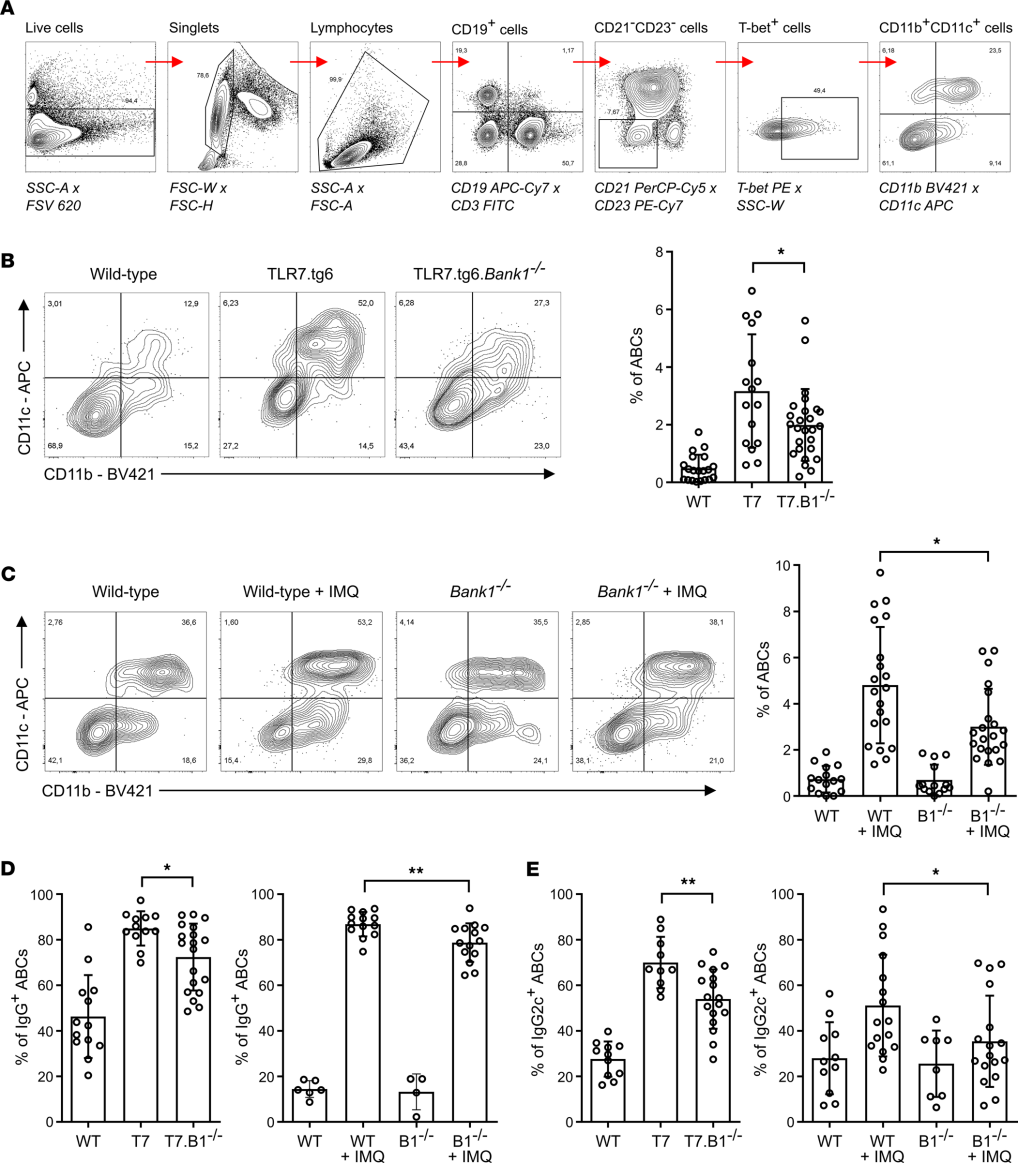
Age-associated B cells are reduced in the absence of *Bank1* in TLR7.tg6 and IMQ-treated mice. (**A**) Gating strategy to detect ABCs by flow cytometry. Sequential gating including: FSV620 live cells; FSC-W × FSC-H to gate single cells; SSC-A × FSC-A to gate lymphocytes; finally, ABCs were CD19^+^CD3^–^CD21^–^CD23^–^T-bet^+^CD11b^+^CD11c^+^ cells. (**B**) Frequency of ABCs among CD19^+^ B cells from the spleens of the TLR7.tg6 model. Total mice analyzed: WT (*n* = 20), T7 (*n* = 16), T7.B1^–/–^ (*n* = 26). (**C**) Frequency of ABCs among CD19^+^ B cells from the spleens of the IMQ-induced model. Total mice analyzed: WT (*n* = 15), WT + IMQ (*n* = 20), B1^–/–^ (*n* = 14), B1^–/–^ + IMQ (*n* = 21). (**D**) Frequency of IgG^+^ cells among ABC population from the spleens of TLR7.tg6 and IMQ-induced models. Total mice analyzed: WT (*n* = 13), T7 (*n* = 12), T7.B1^–/–^ (*n* = 19); and WT (*n* = 6), WT + IMQ (*n* = 13), B1^–/–^ (*n* = 4), B1^–/–^ + IMQ (*n* = 14). (**E**) Frequency of IgG2c^+^ cells among ABC population from the spleens of TLR7.tg6 and IMQ-induced models. Total mice analyzed: WT (*n* = 11), T7 (*n* = 10), T7.B1^–/–^ (*n* = 17); and WT (*n* = 10), WT + IMQ (*n* = 16), B1^–/–^ (*n* = 6), B1^–/–^ + IMQ (*n* = 17). Each point represents 1 mouse. Data are shown as mean ± SEM. Mann-Whitney *U* test with Welch’s correction was used to test statistical significance.

ABCs were characterized according to previous studies (18, 19) as CD19^+^CD21^–^CD23^–^CD11b^+^CD11c^+^T-bet^+^ B cells. The gating strategy is shown in Figure 3A.

A significant increase in the frequency of ABCs was observed in TLR7.tg6 mice by 32 weeks of age and in IMQ-treated WT mice by 20 weeks compared with age-matched healthy controls (Figure 3, B and C). This population was barely detected at earlier weeks in WT mice. Spleens of 10-week-old TLR7.tg6 mice displayed a slight enlargement with an ABC frequency already significantly higher in comparison with 10-week-old WT mice (Supplemental Figure 2, A and B). In TLR7.tg6.*Bank1^–/–^* and IMQ-treated *Bank1^–/–^* mice, the frequency of ABCs was significantly reduced compared with *Bank1*-sufficient lupus-prone mice (Figure 3, B and C). Additionally, to confirm the role of *Bank1*, through TLR7-dependent pathway, in the control of the ABC population, we evaluated the percentage of ABCs in a third lupus model, the B6.*Sle1*.*yaa*. An accumulation of ABCs was observed in B6.*Sle1*.*yaa*.*Bank1^+/+^* with a significant reduction in *Bank1*-KO animals (Supplemental Figure 2C). Taken together, these results suggest that aberrant expansion of ABCs in *Tlr7*-dependent SLE mice is limited by *Bank1* deficiency.

We next determined the frequency of IgG^+^ and IgG2c^+^ ABCs. In both lupus models, mice deficient in *Bank1* showed a significantly reduced proportion of IgG^+^ ABCs compared with TLR7.tg6 and IMQ-treated WT mice (Figure 3D). Furthermore, the proportion of IgG2c^+^ ABCs was also significantly decreased in TLR7.tg6.*Bank1^–/–^* and IMQ-treated *Bank1^–/–^* mice compared with *Bank1*-sufficient lupus-prone mice (Figure 3E). Thus, *Bank1* deficiency reduces the generation and expansion of IgG^+^ and IgG2c^+^ ABCs in our lupus models.

The induction of ABCs can be triggered through the activation of TLR7 and IL-21 signaling pathways (19, 25). Thus, we directly examined the capacity of these stimuli to promote in vitro ABC differentiation in B cells derived from young WT and *Bank1^/–^* mice. The addition of IMQ, alone or in combination with IL-21, led to the differentiation toward ABCs. However, the deficiency of *Bank1* did not result in a significant reduction in the appearance of these cells (Supplemental Figure 2D), suggesting that *Bank1* could affect a B cell population at a stage not stimulated in vitro.

### Single-cell RNA-Seq analysis identifies ABCs as a unique and distinct B cell population.

**Figure 4:**
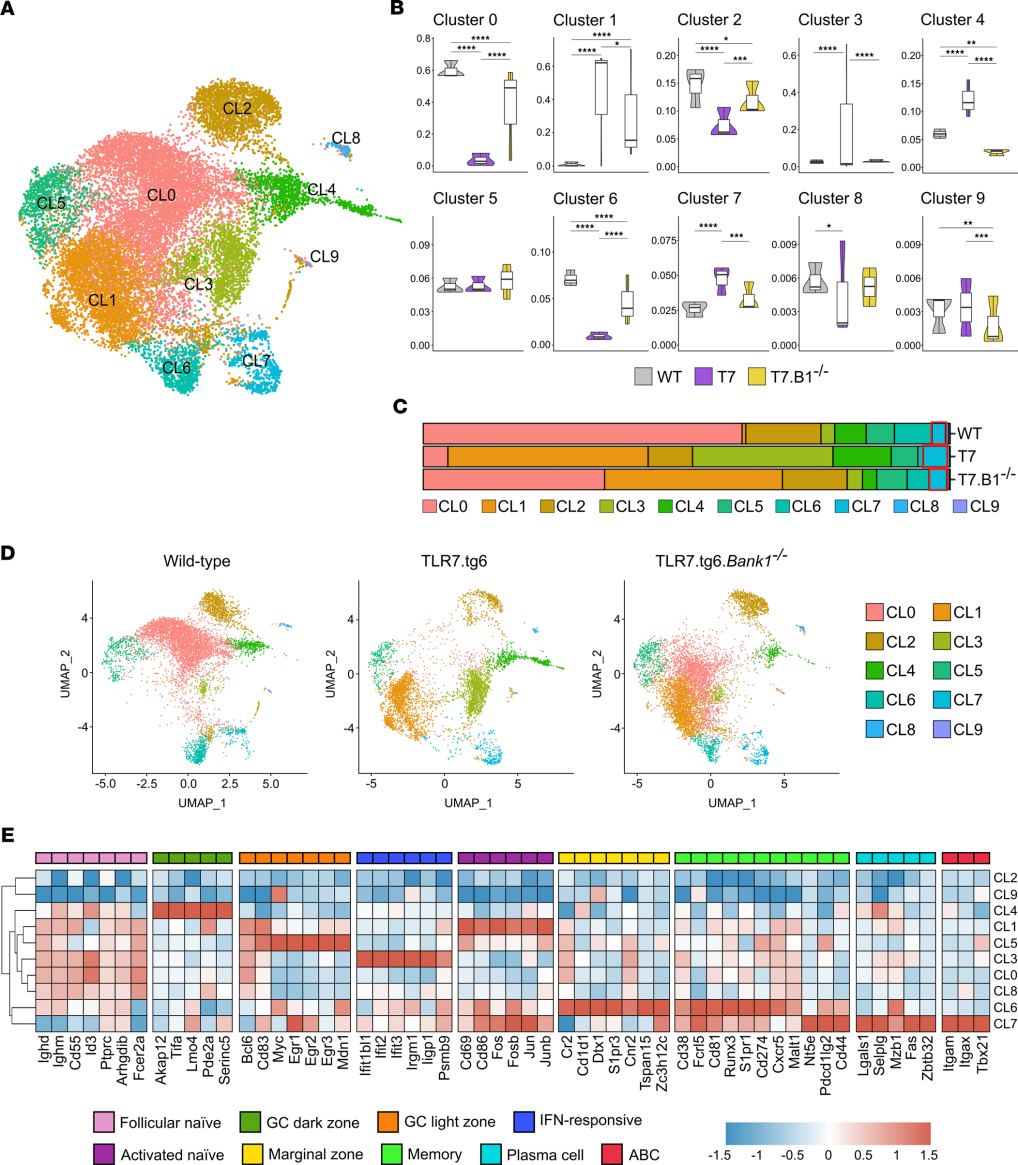
Clustering of B cells from scRNA-Seq analysis. (**A**) Two-dimensional UMAP projection of scRNA-Seq data showing clusters of B cells among total splenocytes from the TLR7.tg6 model. Total mice analyzed: WT (*n* = 3), T7 (*n* = 3), T7.B1^–/–^ (*n* = 3). (**B**) Bar plot representing the cell abundance of each cluster across samples and conditions (WT, TLR7.tg6, and TLR7.tg6.*Bank1^–/–^*). (**C**) Proportion of cells belonging to each cluster per group. Colors represent the different B cell clusters. (**D**) Individual UMAPs from each condition. (**E**) Heatmap showing the expression of the most characteristic markers from each B cell subpopulation comparing each cluster against the remaining clusters. Color scale represent the normalized expression by *Z* score.

To better understand the role of *Bank1* in B cell differentiation and activation in the context of lupus inflammation, a single-cell RNA-Seq (scRNA-Seq) analysis was performed. Splenocytes from 3 mice of each genotype, WT, TLR7.tg6, and TLR7.tg6.*Bank1^–/–^*, were used for the analysis, obtaining 18 clusters of cells. Gene markers for the main spleen cell types were used to annotate cells (Supplemental Figure 3, A–C). Cells annotated as B cells, based on the expression of the pan–B cell genes *Cd19*, *Cd79a*, and *Ms4a1* (clusters 0 and 5), were selected and the entire pipeline reanalyzed. A total of 10 clusters of B cells were obtained (Figure 4A). The relative proportion of cells in each cluster was displayed for each group of mice, and statistical significance was determined by comparing the proportions of each cluster across groups by paired comparison (Figure 4, B–D). These B cells were annotated as FO naive B cells, germinal center dark and light zone B cells, IFN-responsive B cells, naive activated B cells, MZ B cells, MBCs, PCs, and possible ABCs, using gene-markers according to known annotations (30–32) (Figure 4E). Three clusters, marked as CL2, CL8, and CL9, were highly heterogeneous and their annotation was not possible. Furthermore, these clusters were clearly disconnected from all others. Consequently, they were excluded from further analysis and descriptions. Only CL7 was composed of markers compatible with ABCs. This ABC cluster exhibited significant enrichment in TLR7.tg6 mice in comparison with WT mice. The proportion of ABC CL7 was significantly reduced in TLR7.tg6.*Bank1^–/–^* mice (Figure 4, B–D).

**Figure 5:**
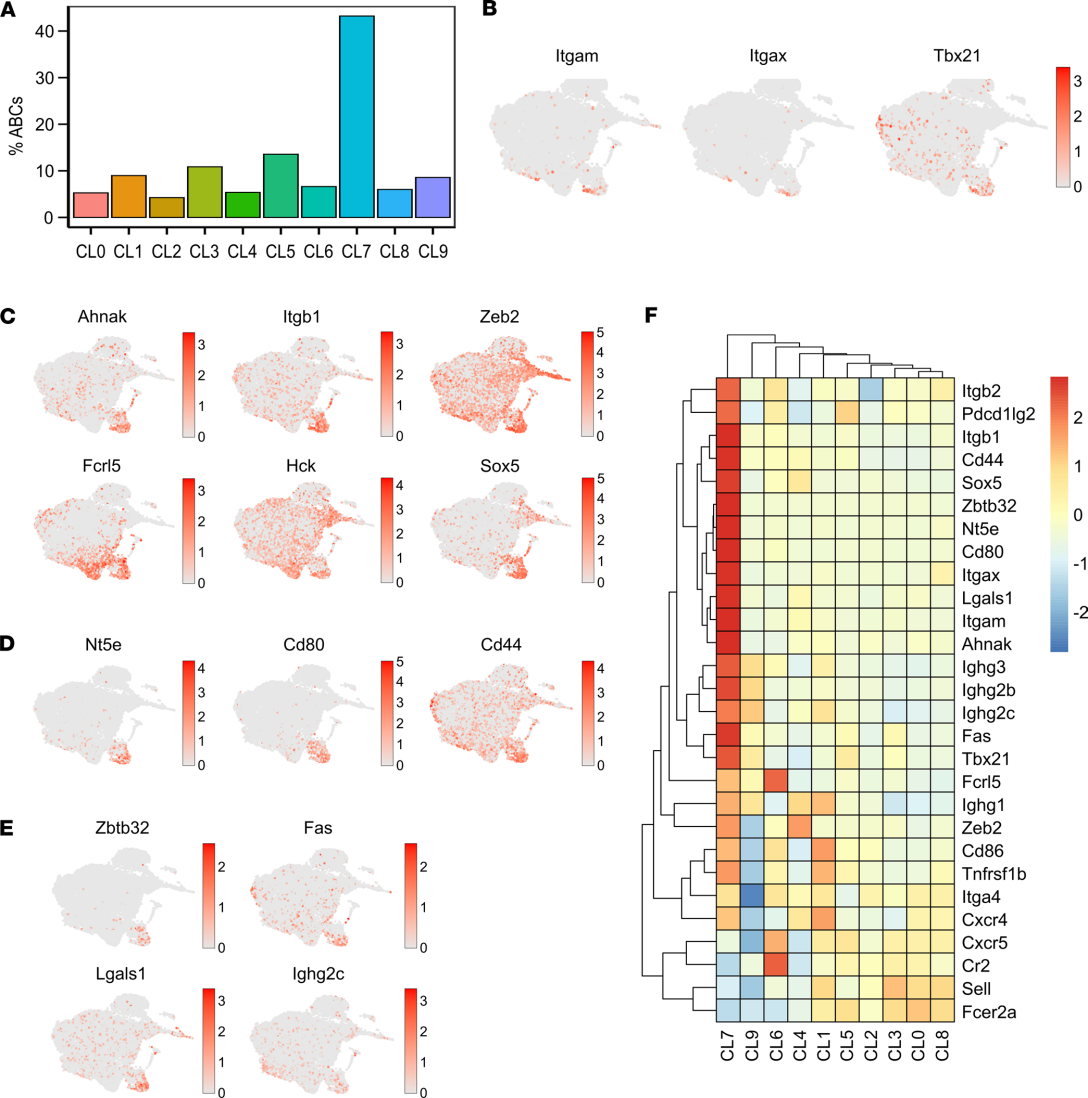
scRNA-Seq analysis identifies ABCs as a unique and distinct B cell population. (**A**) Scoring obtained using AddModuleScore function from Seurat for genes used for ABC isolation (*Tbx21*, *Itgax*, and *Itgam*) in each cluster. (**B**) Individual UMAPs showing the expression levels of typical markers of ABCs. (**C**–**E**) Individual UMAPs showing the expression levels of typical markers of: ABCs and atypical memory B cells (atMBCs) (**C**), memory B cells (MBCs) (**D**), and plasma cells (PCs) (**E**). (**F**) Heatmap displaying selected significantly differentially expressed genes with log_2_ fold-change > 1 (log_2_FC), between each cluster. Color scale represent the normalized expression by *Z* score.

This ABC population exhibited a unique gene expression profile, which was distinct from that of other B cell populations (Supplemental Data File 1). In addition to the known markers, including *Tbx21* (T-bet), *Itgax* (CD11c), and *Itgam* (CD11b) (Figure 5, A and B), *Ahnak*, *Itgb1*, *Hck* and *Zeb2* — which have been identified as ABC markers in recent studies (33–36) — were overexpressed (Figure 5C and Supplemental Data File 1). Interestingly, these genes have been also categorized into a subtype of MBCs referred to as atypical MBCs (atMBC). CL7 exhibited the significant enrichment of other genes commonly observed in atMBCs, including *Sox5*, *Fcrl5*, and *Tnfrsf1b* (36, 37) (Figure 5C and Supplemental Data File 1). Also, genes associated with a MBC profile, such as *Nt5e*, *Cd80*, and *Cd44*, were predominantly expressed in CL7 (Figure 5D and Supplemental Data File 1), implying that what is defined as ABCs may possess a MBC-like phenotype that distinguishes them from classical MBCs (36, 38). Furthermore, this cluster had the increased expression of genes that facilitate B cell activation and the differentiation into PCs, such as *Fas*, *Zbtb32*, and *Lgals1* (35, 36, 39), suggesting that the cells within could be producing antibodies. Additionally, although barely detected, the presence of the *Ighg2c* gene, which encodes the IgG2c isotype, was observed in CL7 (Figure 5E and Supplemental Data File 1). Other IgG subclass immunoglobulin genes were found within this cluster, such as *Ighg1*, *Ighg2b*, and *Ighg3*. All these IgG heavy chain genes were specifically upregulated in CL7 as compared with other clusters (Figure 5F and Supplemental Data File 1). As expected, expression of *Cr2* (CD21) and *Fcer2a* (CD23) genes was downregulated in this cluster (Figure 4E and Supplemental Data File 1).

Separate from *Itgb1* (VLA-4 β subunit), CL7 exhibited the presence of specifically expressed genes that encode for integrins, including *Itga4* (VLA-4 α subunit) and *Itgb2* (LFA1) (Figure 5F and Supplemental Data File 1). These integrins play crucial roles in cell adhesion and migration by interacting with VCAM-1 and ICAM-1, respectively. As these ligands are expressed within the spleen MZ (40), the potential retention of these ABCs in this location is suggested. *Cxcr4* was also highly expressed in this cluster, along with a reduced expression of *Cxcr5* and *Sell* (Figure 5F and Supplemental Data File 1), implying a possible end of these cells in the extrafollicular area (41).

Nevertheless, we reasoned that the heterogeneous pattern of ABCs could be due to the presence of subclusters of cells in different transition stages from having been ABCs (e.g., PCs) or to be ABCs (e.g., MBCs or activated B cells). We thus performed a subclustering of CL7, and we observed 2 subclusters named subCL7-0 and subCL7-1 (Supplemental Figure 4A). SubCL7-0 primarily encompassed ABCs, with the expression of classical and recently discovered ABC-linked markers, exhibiting a gene expression pattern also associated with atMBCs (e.g., *Itgam*, *Itgax*, *Tbx21*, *Zeb2*, *Itgb1*, *Hck*, *Ahnak*, and *Sox5*) (Supplemental Figure 4, B and C). This cluster also exhibited the same MBC markers, *Nt5e*, *Cd44*, and *Cd80* (Supplemental Figure 4D). The expression of the PC genes was also elevated in subCL7-0 (e.g., *Zbtb32*, *Cd86*, *Fas*, *Lgals1*, and *Ighg2b*), and their presence within the ABC area was corroborated with new Uniform Manifold Approximation and Projection (UMAP) visualizations (Supplemental Figure 4E). *Ighg2c* was barely detectable, although positive cells sharing the ABC area were observed. On the contrary, *Ighg1* and *Ighg3* were almost absent in the entire cluster. *Cxcr4* expression was also elevated in this cluster and colocalized in the same area (Supplemental Figure 4F). Conversely, subCL7-1 cells presented a more heterogeneous expression profile, including some markers suggestive of naive (e.g., *Sell* and *Id3*), activated naive (e.g., *Mpp6*), prememory and MBCs (e.g., *Ccr6* and *Cotl1*), markers linked with ABCs (*Hopx*), PCs (*Cd180*), and even markers associated with both proinflammatory (e.g., *S100a6* and *Drd3*) and antiinflammatory (e.g., *Sh3bp5*) responses (Supplemental Data File 2). Due to the limited number of cells identified after subclustering, additional interpretation of these cells in relation to genotypes was not feasible.

These results suggest that CL7 is enriched in ABC markers, characterized by a distinct profile and phenotype, closely resembling atMBCs. Still, ABCs in subCL7-0 could represent a transitional state of memory cells en route to becoming fully differentiated long-lived PCs, while subCL7-1 comprises a variety of transitional ABCs at stages yet to be defined. Our results, however, support CL7 as a full ABC cluster on which we analyzed further.

### Bank1 deficiency induces a distinctive transcriptional profile in ABCs.

We next compared the transcriptomes of CL7 in WT, TLR7.tg6, and TLR7.tg6.*Bank1^–/–^* B cells, revealing a number of genes showing differential expression across the 3 groups (Supplemental Figure 5 and Supplemental Data File 3). While TLR7.tg6 CL7 cells exhibited higher expression of genes related to a proinflammatory response (e.g., *S100a8*, *S100a9*, *Cirbp*, and *Rbm3*) and expressed the immunoglobulin transcripts for IgG1 (*Ighg1*) and IgG2c (*Ighg2c*), TLR7.tg6.*Bank1^–/–^* CL7 cells exhibited higher expression of genes related with an antiinflammatory response (e.g., *Sik1*, *Pdcd4*, *P2ry10*, *Zc3h12d*, *Zfp36l2*, *March1*) and heavy chain genes changed to the IgG3 (*Ighg3*) isotype. Interestingly, several genes whose expression is mediated by IFNs were particularly upregulated in TLR7.tg6 CL7 cells (e.g., *H3f3b*, *H2-ab1*, and *Camp*) as compared with TLR7.tg6.*Bank1^/–^* CL7 cells. Additionally, TLR7.tg6.*Bank1^–/–^* CL7 cells also upregulated genes related with control of protein transport and transcriptional repression (Supplemental Figure 5). Thus, *Bank1* alters the proinflammatory phenotype of ABCs, which in its absence become antiinflammatory.

### TLR7.tg6 mice exhibit a second cluster of B cells strongly modulated by Bank1 that show increased expression of IFN-stimulated genes.

Differentially expressed gene (DEG) analysis comparing clusters and groups was performed focusing on the top 10 genes most differentially expressed comparing each cluster with the rest of the clusters (Supplemental Figure 6A and Supplemental Data File 3). In terms of number of genes, the main differences between TLR7.tg6 and TLR7.tg6.*Bank1^/–^* mice appeared in clusters 4, 3, and 1 (Supplemental Figure 6B), and the frequency of clusters 4 and 3 strongly increased in TLR7.tg6 mice compared with TLR7.tg6.*Bank1^/–^* mice.

In comparison with the TLR7.tg6.*Bank1^–/–^*, the TLR7.tg6 group displayed a higher frequency of cluster 4 (Figure 4, B–D). This cluster could be encompassing cells related to a GC dark zone due to the expression of *Akap12*, *Tifa*, *Lmo4*, *Pde2a*, and *Serinc5*. CL4 exhibited genes related with transitional B cells (e.g., *Vpreb3*, *Sox4*, and *Tnfrsf13c*) (Figure 4E, Supplemental Figure 6A, and Supplemental Data File 1). In TLR7.tg6 mice, CL4 showed high expression of genes involved in the humoral response and immunoglobulin production, such as *Ighg1* and a PC surface marker, *Slamf7*, as well as inflammatory response genes (e.g., *Iigp1*, *Irf4*, *S100a8*, and *S100a9*). In contrast, TLR7.tg6.*Bank1^–/–^* CL4 cells displayed high expression of *Swap70*, known to limit the generation of ABCs in response to IL-21 (25). This cluster also had upregulated expression of antiinflammatory (e.g., *Btla*, *Tsc22d3*, *Zfp36l2*, and *Pdcd4*) and proinflammatory transcripts (e.g., *H2-aa* and *Dennd4a*) (Supplemental Data File 3). Thus, we infer that CL4 represents a subpopulation of B cells within the GC’s dark zone, with proliferative and proinflammatory capabilities, reduced in *Bank1-*deficient mice.

Cluster 3 exhibited the most significant variation in gene expression, with the TLR7.tg6 group showing the highest enrichment compared with the TLR7.tg6.*Bank1^–/–^* group (Figure 4, B–D). This cluster comprised B cells enriched in IFN-stimulated genes (ISGs), with many of them featuring in the top 10 DEGs between clusters (e.g., *Irgm1*, *Iigp1*, *Psmb9*, *Ifi206*). Other ISGs exclusive of CL3 included *Ifit1bl1*, *Ifit2*, and *Ifit3* (Figure 4E, Supplemental Figure 6A, and Supplemental Data File 1). TLR7.tg6 CL3 cells exhibited genes with an IFN signature and the potential for a proinflammatory response (e.g., *Ifgga4*, *Ffar2*, *Iigp1c*, *Iigp1*, *Cdk8*). Furthermore, genes involved in positive regulation of cytokine production and humoral response mediated by immunoglobulin production were observed in this group (e.g., *Shas3*, *Ptpn6*, *Tnf*, *Slamf1*, *B2m*, *Pycard*, *Cd48*), as well as genes with an antigen presentation role (e.g., *H2-dma*, *H2-dmb2*, *H2-oa*). Among the top 10 DEGs in TLR7.tg6.*Bank1^–/–^* CL3 cells, the *Cytip* gene (or *Cybr*) is particularly noteworthy due to its ability to inhibit T-bet and IFN-γ expression through p38 inhibition (42). Additionally, several genes in this group have a described antiinflammatory function and can block the proinflammatory NF-κB pathway (e.g., *Tsc22d3*, *Rnf213*, *Trim30a*). There were also genes exhibiting increased expression following B cell activation through surface BCR and/or CD40 receptor (e.g., *Junb*) or induced by IFN activation (e.g., *Dennd4a*, *Ccnd3*). This group also contains genes involved in cytokine and IFN responses (e.g., *Oasl1*, *Oas2*, *Stat4*, *Irf7*, *Ifitm3*, *Myc*) and genes implicated in negative regulation of IFN and innate immune responses (e.g., *Parp14*, *Lgals9*, *Oas3*) (Supplemental Data File 3). These findings suggest that CL3 encompasses a unique population of ISG-expressing B cells, clearly reduced in the absence of *Bank1*. However, in contrast with the ABC cluster, *Bank1* deficiency did not necessarily affect the expression of certain ISGs.

### ABCs arise from a B cell cluster with MZ and memory cells phenotype.

**Figure 6:**
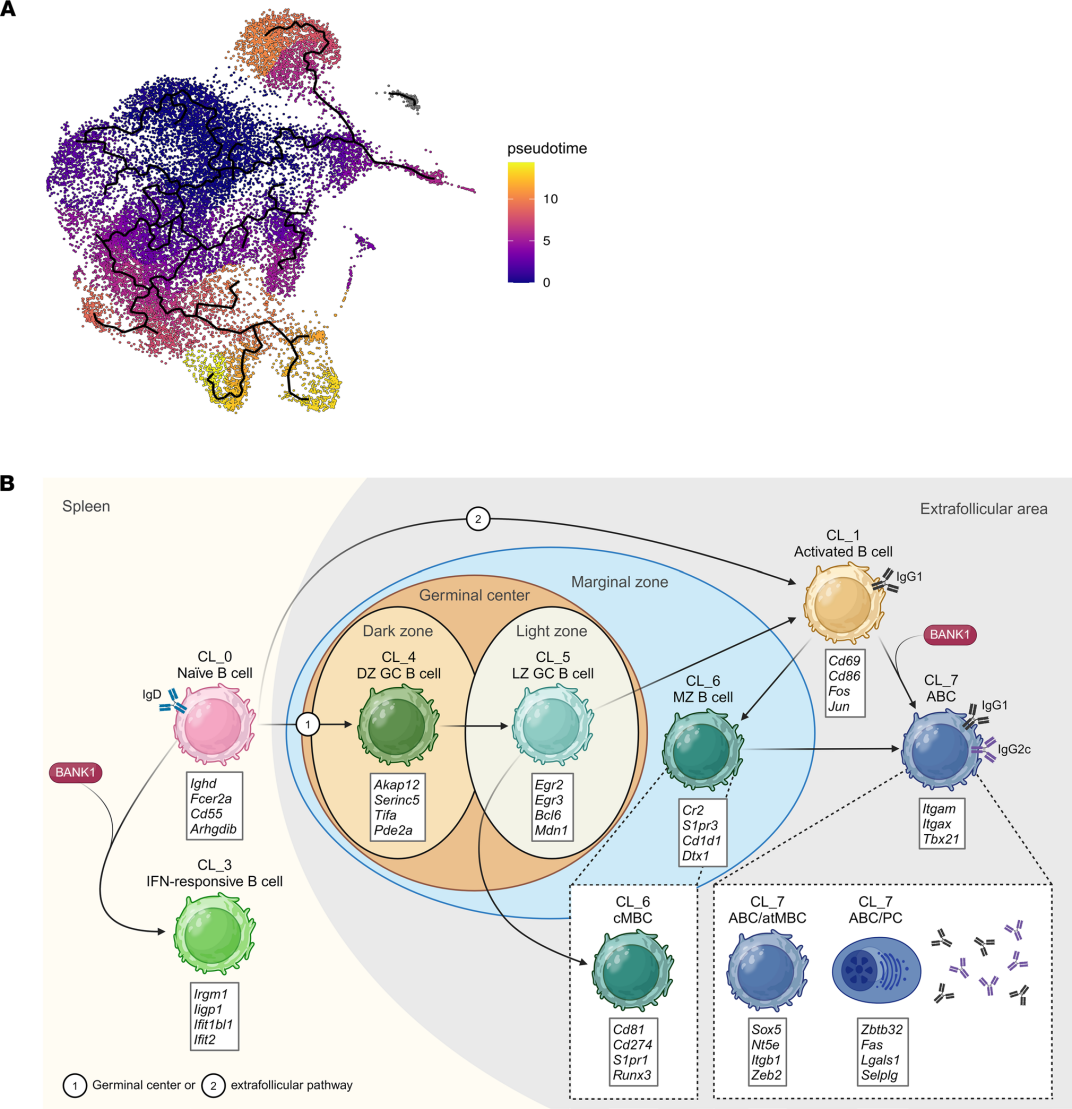
Differentiation trajectory toward ABC cluster 7. (**A**) UMAP visualization of the clusters arranged along trajectories, with CL0 as initial pseudotime (root), colored by inferred pseudotime, calculated by Monocle 3. (**B**) Illustration of the different stages of B cell subpopulation development leading to the formation of ABCs CL7 within the spleen, based on prior trajectory analysis. Arrows indicate subpopulation differentiation steps. Maroon circles labeled “BANK1” indicate *Bank1*’s role in differentiation, or where the frequency of the following B cell cluster is diminished due to *Bank1* deficiency. Numbers denote 2 possible differentiation pathways: germinal center (labeled 1) or extrafollicular pathway (labeled 2). Each cluster is accompanied by a box below, listing the most characteristic and upregulated genes within each subpopulation. DZ GC, dark zone germinal center; LZ GC, light zone germinal center; MZ, marginal zone; cMBC, classic memory B cell; ABC/atMBC, age-associated B cell/atypical memory B cell; ABC/PC, age-associated B cell/plasma cell.

To gain comprehensive understanding of the pathway associated with the transition toward the CL7 ABCs, we conducted a pseudotime trajectory analysis (Figure 6A). Initially, CL0 was selected as the root element due to its phenotype, characterized as FO naive B cells. The analysis revealed a progressive transition from CL0 cells along 2 distinct developmental trajectories. One of these trajectories clearly suggested that CL3 B cells originated directly from CL0 but did not reach CL7. The other trajectory originated in CL0 (root) and continued through clusters 1, 6, and ultimately 7. Differentiation trajectories are schematized in Figure 6B. As an alternative hypothesis, despite the infeasibility of CL2 annotation, we conducted the same trajectory analysis selecting this cluster as the initial pseudotime. This selection was based on the hypothesis that cluster 2 (CL2) might represent a stage of immature B cells, supported by the expression of *Pax5* and *Bcl11a*. In this model, the subsequent phases of B cell differentiation would progress through CL4, acting probably as transitional B cells, and finally CL0, corresponding to FO mature naive B cells (Supplemental Figure 7 and Supplemental Data File 1).

Cells from CL0 were more frequently present in WT and TLR7.tg6.*Bank1^–/–^* mice than in TLR7.tg6 mice (Figure 4, B–D). CL0 had high expression of *Ighd* and *Fcer2a*, representing mature FO naive B cells, high expression of *Arhgdib* and *Cd55*, and overexpression of genes essential for cell survival and anti-inflammatory features (e.g., *Id3*, *Emp3*, *Gimap1*, *Sh3bgrl3*) (Figure 4E, Supplemental Figure 6A, and Supplemental Data File 1). These results indicate that *Bank1* deficiency normalized the presence of FO naive B, which were nearly absent in lupus-prone mice.

CL5 appeared to have similar frequencies in TLR7.tg6 and TLR7.tg6.*Bank1^–/–^* mice (Figure 4, B–D). CL5 B cells presented upregulated genes involved in GC reactions, specifically related with the light zone (e.g., *Egr1*, *Egr2*, *Egr3*, *Mdn1*); in activation (e.g., *Bcl2a1b*, *Mif*, *Myc*); and in BCR-mediated proliferation (e.g., *Ccnd2*, *Il4i1*, *Marcksl1*). Furthermore, CL5 exhibited higher expression of the *Bcl6* gene, a critical transcription factor essential for GC formation, compared with clusters 6 and 7 (Figure 4E, Supplemental Figure 6A, and Supplemental Data File 1).

The size of CL1 was augmented in lupus-prone mice compared with WT mice and was slightly increased in TLR7.tg6 compared with TLR7.tg6.*Bank1^–/–^* mice (Figure 4, B–D). CL1 comprises genes involved in B cell activation (e.g., *Cd69*, *Cd86*, *Fos*, *Fosb*, *Jun*, *Junb*, *Uba52*) (Figure 4E, Supplemental Figure 6A, and Supplemental Data File 1). Interestingly, CL1 displayed higher expression of *Cxcr4* and *Ighg1* genes, closely resembling the expression observed in CL7, suggesting a potential localization of these cells in the extrafollicular area (Supplemental Data File 1). TLR7.tg6 CL1 cells exhibited higher expression of genes of the major histocompatibility complex class II (MHC-II) antigen presentation (e.g., *H2-Aa*, *H2-Ab1*). Furthermore, this group was enriched in functions related to humoral responses mediated by immunoglobulin production and displayed elevated expression of *Ighg1* and *Ighg2c* genes compared with *Bank1*-deficient mice (Supplemental Data Files 3–5). In contrast, TLR7.tg6.*Bank1^–/–^* CL1 cells exhibited significant upregulation of antiinflammatory function genes, suppressing proliferation, cytokine secretion, and surface expression of MHC-II (e.g., *Btla*, *March1*, *Dnaja1*, *Arhgap15*) (Supplemental Data Files 3–5). Thus, *Bank1* deficiency partially normalized the frequency of CL1 activated B cells, potentially contributing to the regulation of immune responses.

CL6 is primarily composed of MBCs. The TLR7.tg6.*Bank1^–/–^* group, as well as the WT group, exhibited notably higher frequencies of this cluster than the TLR7.tg6 group (Figure 4, B–D). CL6 exhibited genes found in MBCs in mice and humans (e.g., *Cxcr5*, *S1pr1*, *Malt1*, *Runx3*) and only in mice (e.g., *Cd274*, *Cd81*). Interestingly, CL6 also shared genes of MBCs and atMBC, such as *Cd38* and *Fcrl5,* respectively, with CL7 ABCs. Additionally, CL6 highlights genes that are characteristic of MZ B cells — namely *Dtx1*, *Cd1d1*, and *Cr2*. The expression of genes encoding receptors involved in MZ migration, such as *S1pr3* and *Cnr2*, was also observed (43, 44) (Figure 4E and Supplemental Data File 1). CL6 included the *Atxn1* gene as a transcriptional repressor that modulates the expression of the other memory markers *Cd80* and *Cd44* (both among the top 10 genes in CL7) (Supplemental Figure 6A and Supplemental Data File 1). Interestingly, TLR7.tg6.*Bank1^/–^* CL6 cells exhibited a significant upregulation of genes linked to antiinflammatory functions (e.g., *Kctd2*, *Serpine2*, *Pdcd4*, *Zfp36l2*, *Sesn1*) and a lesser variety of upregulated genes related to proinflammatory functions (e.g., *Ccl5*, *Map3k9*) (Supplemental Data File 3). Based on these observations, it can be inferred that the deficiency of *Bank1* impedes the differentiation process from CL6 to CL7, leading to an increase in the subset of MZ B cells and MBCs. Conversely, in the TLR7.tg6 group, this transition is facilitated, thus increasing the number and percentage of CL7 ABCs.

### Numbers of ABCs closely correlate with Th cells in the extrafollicular space where Bank1 modulates IgG2c production.

The analysis of scRNA-Seq data revealed that activated B cells (CL1) and ABCs (CL7) displayed elevated expression of the *Cxcr4* gene relative to the remaining clusters (Supplemental Data File 1). This finding implies a plausible localization of these populations within the extrafollicular cell niche. Additionally, ABCs exhibited a PC phenotype (Figure 5E and Supplemental Data File 1), suggesting their final destiny as autoantibody-producing cells in the autoimmune process. Hence, we wanted to determine the effect of *Bank1* deficiency on both the expression of *Cxcr4* and the cellular localization of ABCs outside the GCs.

**Figure 7:**
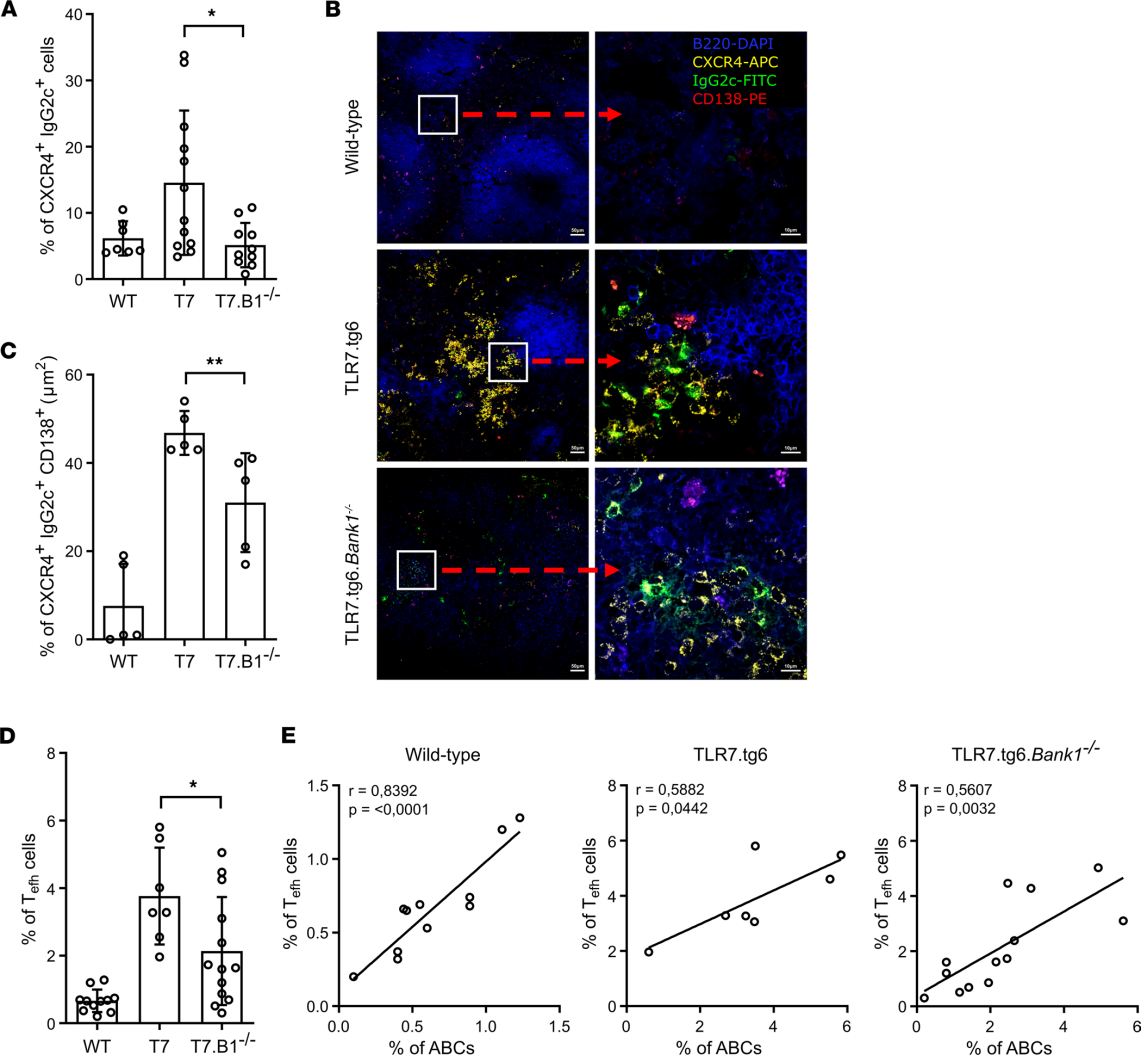
Numbers of ABCs closely correlate with Th cells in the extrafollicular space where *Bank1* modulates IgG2c production. (**A**) Frequency of CXCR4^+^IgG2c^+^ cells among CD138^+^B220^–^ cells from the spleens of the TLR7.tg6 model. Total mice analyzed: WT (*n* = 7), T7 (*n* = 12), T7.B1^–/–^ (*n* = 10). (**B**) Representative cryosections of spleens from WT, TLR7.tg6, and TLR7.tg6.*Bank1^–/–^* mice, stained with anti-B220 DAPI, anti-CXCR4 APC, anti-IgG2c FITC, and anti-CD138 PE. The red arrow shows a digital zoom (×2) selected from each image. Scale bar: 100 μm (left panels); 20 μm (right panels). This representative experiment was conducted 3 different times. All images were captured using a Confocal Laser Microscope Zeiss LSM 710. (**C**) Area in μm^2^ of CXCR4^+^IgG2c^+^CD138^+^ cells in the spleens from 32-week-old TLR7.tg6 model. Total mice analyzed: WT (*n* = 5), T7 (*n* = 5), T7.B1^–/–^ (*n* = 5). (**D**) Frequency of Tefh cells (CD62L^–^CD44^+^PDGL-1^–^CXCR4^+^) among CD3^+^CD4^+^ cells from the spleens of 32-week-old TLR7.tg6 model. Total mice analyzed: WT (*n* = 11), T7 (*n* = 7), T7.B1^–/–^ (*n* = 13). (**E**) Correlation between the percentage of ABCs among CD19^+^ B cells and the percentage of Tefh among CD4^+^ T cells, using Pearson correlation coefficient, in WT, TLR7.tg6, and TLR7.tg6.*Bank1^–/–^* mice. Each point represents 1 individual mouse. Data are shown as mean ± SEM. Mann-Whitney *U* test with Welch’s correction was used to test statistical significance.

Autoantibody-producing cells can be generated via GC and extrafollicular pathways (45). We observed an increased dysregulation in the formation of GCs within the TLR7.tg6 group, characterized by an expanded dark zone and a disrupted GC structure. On the other hand, *Bank1* deficiency restored the GC structure to one more closely resembling that of healthy mice (Supplemental Figure 8A). In TLR7.tg6 mice, the frequency of extrafollicular B cells expressing a PC phenotype (B220^+^CD138^+^IgG2c^+^CXCR4^+^) was significantly higher in comparison with *Bank1*-deficient and WT mice (Figure 7A). Furthermore, the majority of these splenic TLR7.tg6 CXCR4^+^ B cells displayed an expanded distribution out of the GCs being CD138^+^ and IgG2c^+^ (Figure 7B). In TLR7.tg6 mice, CXCR4^+^ cells colocalized with T-bet protein expression (Supplemental Figure 8B), and the frequency of T-bet^+^ extrafollicular B cells was increased (Supplemental Figure 8C). In contrast, *Bank1*-deficient and WT mice had these extrafollicular B cells reduced. Quantification of extrafollicular B cell areas in the spleens showed a statistically significant increase in TLR7.tg6 mice compared with WT and TLR7.tg6.*Bank1^–/–^* mice (Figure 7C). Our results suggest that ABCs are located within the extrafollicular area, where they might serve as pivotal contributors to the production of autoantibodies. Notably, their presence in this specific area is diminished in the absence of *Bank1*.

We have previously shown that *Bank1* deficiency downregulated surface expression of CXCR4 on T FO helper (Tfh) cells in B6.*Sle1*.*yaa* mice (17). CXCR4^hi^ T cells are considered T extrafollicular helper (Tefh) cells, providing help to extrafollicular plasmablast responses in autoimmune-prone mice (41). Therefore, we investigated the possible interplay between ABCs and Tefh cells. Frequency of Tefh cells (CD3^+^CD4^+^CD62L^–^CD44^+^PDGL-1^–^CXCR4^+^) in TLR7.tg6 mice was significantly higher compared with WT and TLR7.tg6.*Bank1^–/–^* mice (Figure 7D). Interestingly, in all groups of mice, the number of ABCs exhibited a significant positive correlation with the number of Tefh cells (Figure 7E). This finding implies a potential interaction at the T-B border between both cell types.

## Discussion

In the present work, we confirmed our previous results using the B6.*Sle1*.*yaa* model with and without *Bank1*. The major effect that *Bank1* deficiency had was an important reduction in the production of total IgG and IgG autoantibodies. Clearly, the deficiency also had effects on various cell populations. Here, we do take a step further, in the analysis of the ABC population not studied previously in relation to the *Bank1* deficiency. We therefore confirm the increase of ABCs in the 3 lupus strains and how *Bank1* deficiency reduces this population of cells. However, we did not observe changes when performing in vitro differentiation of ABCs derived from naive B cells from the *Bank1*-deficient mice. The reasons for this are unclear, as it is possible, as we observed in the single-cell experiment, that ABCs are derived from memory or pre-MBCs that were probably not stimulated in vitro.

For this reason, we performed an in-depth analysis of gene transcription of B cells at a single-cell level to study the extent to which *Bank1* deficiency affects the ABC population and other B cells during an autoimmune process, drawing the following conclusions:

ABCs are a unique B cell population identified both through traditional and recently discovered ABC markers observed in the scRNA-Seq analysis. In patients with SLE and in lupus-prone mice, ABCs were found to have a PC-like phenotype, capable of producing autoantibodies (25, 46). Consequently, *Bank1* deficiency may impede the differentiation of ABCs toward this particular phenotype by reducing the presence of class-switched IgG and IgG2c ABCs by downregulating *Aicda* (17) and genes associated with features of immunoglobulin-mediated immune responses, including *Zeb2* and *Zbtb32* (35, 39).

In the absence of *Bank1*, ABCs expressed other genes, leaning toward an antiinflammatory phenotype. This is of great interest and suggests that *Bank1* is driving and regulating proinflammatory features and functions of the ABCs and mediating the TLR7-dependent response during autoimmunity, possibly through its interaction with MyD88 and TRAF6 (10). Importantly, these observations align well with the proportion of CL3 IFN-producing B cells, which were extremely reduced in healthy and *Bank1*-deficient mice compared with *Bank1*-sufficient mice. Nevertheless, the exact relationship between CL3 and CL7 remains incompletely understood, as scRNA-Seq trajectory analysis did not suggest any proximity between CL7 ABCs and CL3 IFN cells.

The trajectory analysis revealed the most plausible path for the differentiation of splenic ABCs, clearly pointing toward CL0, 1, and 6, with the latter expressing genes compatible with a MZ and MBC phenotype. In the absence of *Bank1*, CL6 is increased, in what appears to be a retention of these cells in the spleen, affecting also B cell differentiation upstream of this CL6 — that is, CL1. According to current evidence, ABCs can originate both from within and outside GCs, being most commonly observed extrafollicularly, an observation supported by their expression of specific integrins known to be involved in cell retention in this anatomical region. This process may involve 2 distinct differentiation pathways that differ depending on the context (34, 47). Lymphoma studies have indicated that *Bank1* expression is predominantly found outside GCs, specifically in the mantle zone, where *Bank1* exerts its influence in the extrafollicular areas (48). This observation could explain why *Bank1* has no effect on CL5 frequencies between the 3 groups of mice. Considering this, it is likely that mature naive B cells (CL0) become hyperactivated (CL1) in the context of autoimmunity, acquire an atypical memory-like phenotype (CL6), and subsequently differentiate into ABCs (CL7). These cells may then be retained in the MZ in the absence of *Bank1* (49, 50).

The ABC CL7 comprised 2 subclusters, 1 of which appeared to be more enriched in ABCs proper. This however did not exclude the atMBC phenotype observed in ABCs. On the contrary, this first subcluster included MBC genes, but the experiment did show another subcluster possibly having a larger degree of turnover and containing a variety of transitional cells. Further studies would be required in which these cells are studied at the single-cell level to reveal in more detail the granular stages of development and differentiation of the ABCs once they acquire their characteristic markers.

It is worth mentioning that a recent study conducted a scRNA-Seq study in peripheral blood mononuclear cells from patients with lupus, revealing a distinct cluster of B cells characterized by elevated *BANK1* expression. This cluster shared key features with the murine ABC population identified in CL7. Notably, this human B cell cluster exhibited a phenotype reminiscent of extrafollicular, double-negative switched MBCs, commonly referred to as DN2. Moreover, this cluster demonstrated increased expression of genes observed also in our murine ABC-CL7, including human *ITGAX*, *TBX21*, *ZEB2*, *ZBTB32*, and *CD86* (30).

The well-established significance of extrafollicular responses in mouse models of autoimmunity highlights their contribution to the development of autoimmune diseases (51, 52). CL7 ABCs display several extrafollicular characteristics, including the presence of *Cxcr4* and the absence of *Cxcr5* and *Sell* (41, 46). In addition, this localization could be also influenced by lower *Bcl6* expression. Hence, *Bank1* might play a role in regulating the localization and expansion of these cells within extrafollicular regions. In fact, in support of our observation, a recent study used a KO for *Bcl6* to block the formation of GC B cells in an induced lupus model. Such blocking did not modify the development of lupus in the animals that did present with extrafollicular cells (52). These results align with our trajectory analysis on the origin of CL7 ABCs as extrafollicular cells, appearing directly from CL1 activated B cells through CL6. An extrafollicular response is also observed in severe SARS-CoV-2 infection (53) and arising by a dysregulated activated naive B cell compartment. In patients with COVID-19, heightened extrafollicular B cell responses lead to rapid activation of B cells, resulting in a high proportion of atMBCs and antibody-secreting cells. This extrafollicular response shares similarities with that seen in patients with SLE (49). In addition, it is also possible that *Bank1* is modulating these ABCs when, in its absence, there is an important reduction of total IgG and IgG2c, which may come from both ABCs or extrafollicular B cell plasmablasts (54). Notably, we do observe increased expression of the *Igg* genes in the scRNA-Seq experiment that were primarily overexpressed in the ABC cluster and were significantly reduced in the absence of *Bank1*.

Finally, our analyses of CXCR4 do corroborate the close interaction between ABCs and Tefh cells. Studies have suggested that such interaction may be due to the production of IL-21 and through CD40L and ICOS on Tefh cells (41). Nevertheless, our transcriptome data show that *Il21r* and *Icosl* genes were downregulated on CL7 ABCs (Supplemental Data File 1), suggesting an alternative explanation of this interplay between both subsets. A key function attributed to ABCs is their potential to serve as APCs, preferentially localized at the T-B border in the spleen (35, 50). CL7 ABCs displayed elevated expression of MHC-II genes, alongside *Cd80* and *Cd86*. We found that TLR7.tg6 CL7 ABCs were significantly associated with positive regulation of genes involved in antigen processing and presentation, as well as with positive regulation of T cell activation. We also observed a reduced expression of *Cd80* in *Bank1*-deficient mice (Supplemental Figure 5 and Supplemental Data File 5).

All these findings together suggest a role of *Bank1* in the capability of ABCs to interact with Tefh cells, playing a crucial role in facilitating the ABC response and being activated during autoimmunity. Our data imply that *Bank1* exerts an influence on the formation of extrafollicular foci by modulating the enhanced expression of CXCR4 on B and T cells, a mechanism by which these subpopulations could contribute to the disease.

While ABCs appear to be cells of major importance in autoimmunity, we find that the ISG-expressing B cell population (CL3) is also importantly modulated by *Bank1* and that *Bank1* has an important role in determining the expression of ISGs. Interestingly, these cells were not in the trajectory toward ABCs and appeared as the end-point of a pathway arising directly from CL0 of mature naive B cells. These cells appeared to be poised to go through a proinflammatory pathway in the case of the TLR7.tg6, while being kept antiinflammatory and reduced in the absence of *Bank1*. It is plausible that these cells directly respond to type I IFN (IFN-β) produced by *Tlr7*-stimulated cells. The decrease in their numbers may be linked to a reduction of *Irf7* expression, on which IFN-β production is dependent, attributed to the diminished *Tlr7* signaling pathway observed when *Bank1* is absent (17). One gene of interest observed in *Bank1*-deficient CL3 cells was *Cytip* (*Cybr*) a transcription factor that appears to inhibit T-bet, also observed in clusters 0, 2, and 5, although its function has been primarily studied in human T cells (42). The role of CL3 cells during the autoimmune process is unknown, but it could represent the saturation of a system where cells continue to differentiate without control. Discerning the mechanisms behind the development of these cells will be an important path toward understanding B cell differentiation in autoimmunity.

In summary, it is clear from our results that *Bank1*, a gene associated with lupus and autoimmunity in humans, has a major role in the formation and function of the ABC population, possibly due to its involvement in the TLR7-dependent pathway (10), and may exert an indirect influence, potentially by other signals, in their differentiation. Furthermore, our data suggest a significant involvement of *Bank1* in the presence of B cells expressing ISGs. This involvement may be related to reduced *Irf7* expression and IFN-β production, which is attributed to the diminished *Tlr7* signaling pathway observed when *Bank1* is absent (17). In short, the study of *Bank1* is essential to understand its relationship with viral infections, autoimmunity, and the adequate treatment of disease.

## Methods

### Sex as a biological variable.

Our study exclusively examined male mice because TLR7.tg6 and B6.*Sle1.yaa* animals exhibit an increased expression of the *Tlr7* gene, due to its insertion on the Y chromosome, and the disease manifests solely in males.

### Study design.

This study aimed to define the pivotal role of the *Bank1* gene concerning ABCs, and other main B cell subsets, in the context of autoimmunity. To this end, we utilized SLE mouse models distinguished by an exacerbated *Tlr7* expression, and crossed with the KO for *Bank1*. Serum and spleen samples were collected from each animal at the end of the experiment. All surgical procedures were carried out under aseptic conditions to ensure the fidelity of the experimental data. Sample size was determined based on statistical methods, and on our previous experience with these models. The *n* values used for each experiment, along with the corresponding statistical methodologies, are indicated in the figure legends. We conducted in vivo, ex vivo and in vitro experiments, including survival monitoring, spleen weight measurement, ELISA, flow cytometry analysis, B cell stimulation and differentiation cultures, and immunofluorescence analysis. These experiments aimed to determine the phenotypic and functional alterations within ABCs, and key B cell subpopulations, affected by the deficiency of *Bank1*. In general, for in vivo, ex vivo and in vitro studies, at least 3 independent experiments were conducted with biologically independent samples, corresponding to distinct mice. Furthermore, a scRNA-Seq assay was performed to gain a deeper insight into the path of differentiation of ABCs and to assess the transcriptomic changes induced by *Bank1* deficiency on B cell populations. Transcriptomic data were obtained from single cells sorted from total splenocytes of 3 mice per group: healthy control WT, TLR7.tg6, and TLR7.tg6.*Bank1^–/–^* mice.

### Animal models.

TLR7.tg6 and B6.*Sle1*.*yaa* animals were obtained from Darise Farris from the Oklahoma Medical Research Foundation (OMRF), Arthritis & Clinical Immunology Research Program (Oklahoma City, Oklahoma, USA). B6.*Sle1.yaa* mice carry the SLE-susceptibility locus *Sle1* and the *yaa* locus obtained from the BXSB.*yaa* lupus-prone strain. The *yaa* mutation is the result of a translocation of a portion of the X chromosome, containing the *Tlr7* gene, onto the Y chromosome, resulting in the acceleration of systemic autoimmunity in these animals (55). The TLR7.tg6 strain also reflects this expression in male mice, by an insertion of the *yaa* region in the Y chromosome. This strain exhibits 8–16 copies of the *Tlr7* gene on the Y chromosome, which led to a significant 4- to 8-fold rise in *Tlr7* mRNA levels (28). Both models were crossed with *Bank1^–/–^* mice, already backcrossed more than 9 generations with the C57BL/6J (17). C57BL/6J WT control animals were purchased from The Jackson Laboratory (Lyon, France), and used to maintain C57 genetic background of both strains TLR7.tg6 and *Bank1^–/–^*. All animals were maintained under specific pathogen–free conditions in the animal facilities at University of Granada, Centro de Investigación Biomédica (CIBM, Granada, Spain). For the IMQ-induced model, we followed a previous published protocol (29) with small modifications. Ten- to 12-week-old male mice, C57BL/6J WT and *Bank1^–/–^* were treated with 25 mg of Aldara 5% cream (IMQ, 3M Pharmaceuticals), an agonist of the TLR7 receptor, in the left ear 3 times per week for a total of 8 weeks. At 18–20 weeks of age, treated and untreated control mice were sacrificed. TLR7.tg6, TLR7.tg6.*Bank1^–/–^,* and control C57BL/6J male mice were sacrificed at 30–32 weeks of age. For B6.*Sle1*.*yaa*.*Bank1^–/–^* and B6.*Sle1*.*yaa*.*Bank1^+/+^* male mice, experiments were performed at 36 weeks as previously reported (17).

### ELISA.

Blood was obtained at the indicated final points by cardiac puncture, and sera were collected. Levels of autoantibody anti-dsDNA in serum were determined by homemade ELISA following the previously published protocol (56) using 1:1,000 dilutions. Total antibodies (Sm, IgM, total IgG, and IgG1) were measured in sera with commercial ELISA following the manufacturer’s protocol (eBioscience). IgG2c levels in mouse sera were measured with a commercial ELISA kit following the manufacturer’s instructions (Bethyl). The absorbance was measured with an Infinite M200Pro plate reader (Tecan) at 450 and 570 nm.

### Antibodies and flow cytometry analysis.

Spleens were homogenized mechanically with a 5 mL syringe followed by straining through a 70 μm nylon filter under sterile conditions. RBCs were lysed in ammonium-chloride-potassium lysis buffer (ACK) followed by washing and staining for flow cytometry analysis (1 × 10^6^ cells). BD Horizon Fixable Viability Stain 620 (FVS620, 564996, BD Biosciences) or LIVE/DEAD Fixable Aqua (L34957, Thermo Fisher Scientific) were used to exclude death cells from the analysis. Viability dyes were mixed with cells and incubated during 20 minutes at 4°C. Cells were washed and then incubated for 10 minutes at 4°C with TruStain FcX anti–mouse CD16/32 antibody (clone 93, 101319, BioLegend) to block nonspecific antibody binding. The following antibodies were used for multiparameter flow cytometry: anti–mouse CD19 APC eFluor780 (clone eBio1D3, 47-0193-82), anti–mouse CD23 PE-Cy7 (clone B3-B4, 25-0232-82), and anti–mouse CXCR4 V450 (clone 2B11, 48-9991-82), all from Invitrogen; anti–mouse CD21 PerCP-Cy5.5 (clone 7E9, 123415), anti–mouse CD11b BV421 (clone M1/70, 101235), anti–mouse T-bet PE-Cy7 (clone 4B10, 644823), anti–mouse CD3 FITC (clone 17A2, 100203), anti–mouse B220 V500 or anti–mouse B220 PE (clone RA3-6B2, 103247 or 103208), anti–mouse CD138 PE (clone 281-2, 142503), anti–mouse TCR PerCpCy5.5 (clone H57-597, 109227), anti–mouse CD62L PerCP-Cy5.5 (clone MEL-14, 104431), and anti–mouse CD44 PECy7 (clone IM7, 103029), all from BioLegend; anti–mouse IgG2c FITC (ab97254) and anti–mouse IgG (H+L) FITC (ab6785), all from Abcam; anti–mouse T-bet PE (clone 11/41, 561265), anti–mouse CD11c APC (clone HL3, 550261), anti–mouse CD138 BV510 (clone 281-2, 563192), anti–mouse CD4 V500 (clone RM 4-5, 560783), and anti–mouse PSGL1 PE (clone 2PH1, 555306), all from BD Biosciences. For intracellular staining, splenocytes were incubated on a fixation and permeabilization solution (BD Cytofix/Cytoperm Plus; 555028) following manufacturer instructions. In addition to the viability controls, unstained negative controls, single-stained controls, and FMO controls were used to enhance the quality and accuracy of the analysis. Acquisition was performed on a BD FACS Diva or on a Symphony cytometer (BD Biosciences) and analyzed with FlowJo 9.8 (Tree Star Inc.) software.

### Histology and immunofluorescence staining.

Mice were anesthetized and perfused with 20 mL of Dulbecco’s phosphate-buffered saline (DPBS) 1×. Organs were extracted and fixed in paraformaldehyde (PFA) 4% for 12–16 hours at 4°C. Afterward, tissue sections were frozen in an isopentane-dry ice slurry for immunofluorescence in optimal cutting temperature (OCT) and storage at –80°C until sectioning OCT (Tissue-Tek). Sections (8 μm) were fixed 7 minutes in cold acetone (20°C) before being hydrated for 20 minutes in TBS and 20 minutes in TBS with Tween 20 0.05% (TBS-T). Tissue sections were then blocked with TBS-T/BSA 5%, 2 mg/mL of anti–mouse CD16/CD32 (clone 93, 101319, BioLegend), 10% normal rat serum (10710C, Invitrogen), and 10% goat serum (S26100ML, Sigma-Aldrich) for 30 minutes at room temperature. Slides were briefly washed in TBS-T and stained with Fluorescein Peanut Aglutinin–FITC (PNA-FITC) (Vector Laboratories); purified anti–mouse CD169 (MOMA-1) (clone 3D6.112, 142401, BioLegend), followed by goat anti–rat Alexa Fluor 555 secondary antibody (A-21434, Invitrogen); recombinant anti–mouse CD4 antibody (clone EPR19514, ab183685, Abcam), followed by goat anti–rabbit Alexa Fluor 633 secondary antibody (A-21070); anti–mouse CXCR4 (clone 2B11, 14-9991-82, Invitrogen), followed by goat anti–rat APC secondary antibody (A10540, Invitrogen) or CXCR4 APC eFluor 780 (clone 2B11, 47-9991-82, Invitrogen); anti–mouse IgG2c FITC goat (1078-02, SouthernBiotech); anti–mouse B220 ef450 (clone RA3-682, 48-0452-82, Invitrogen); anti–mouse CD138 PE (clone 281-2, 561070, BD Biosciences); and anti–mouse T-bet PE (clone 4B10, 12-5825-82, Invitrogen). Slides were incubated for 45 minutes at room temperature. Cell nuclei were stained with Hoechst 33342 (1 μM, B2261, Sigma-Aldrich) for 5 minutes at room temperature. Slides were mounted with SlowFade Diamond Antifade Medium (S36967, Invitrogen), and images of tissue sections were captured using a Zeiss 710 Laser Scanning Microscope, a Zeiss Plan-Apochromat 20×/0.8 NA objective, and the Zeiss ZEN 2010 software. Fluorescence was acquired sequentially using different lasers for excitation and different photomultipliers for the detection of all fluorescence signals.

### B cell isolation and differentiation in vitro.

Splenocytes from 10-week-old mice were enriched for B cells with a B cell isolation kit (130 090 862, Miltenyi Biotec) following the manufacturer instructions, using the autoMACS Pro Separator (Miltenyi Biotec). B cells were cultured in RPMI medium 1640 (21870076, Thermo Fisher Scientific) supplemented with 10% FBS (S181A, Biowest), 100 U/mL penicillin-streptomycin (15140122, Thermo Fisher Scientific), 1× Non-Essential Amino Acids (11140068, Thermo Fisher Scientific), 2 mM L-Glutamine (A2916801, Thermo Fisher Scientific), 25 mM HEPES (15630 056, Thermo Fisher Scientific), and 50 μM β-Mercaptoethanol (M3148, MilliporeSigma). For stimulation, B cells were treated with 5 μg/mL F(ab’)2-goat anti–mouse IgM (Mu chain, 16-5092-85, Invitrogen) and 5 μg/mL Ultra-LEAF purified anti–mouse CD40 (102811, BioLegend) in the presence or absence of 2.5 μg/mL IMQ (tlrl-imqs, Invivogen), 25 ng/mL mouse recombinant IL-21 (574504, BioLegend), and the combination of both. B cells were collected at 72 hours and analyzed by flow cytometry as described above.

### Single-cell RNA-Seq analysis.

Spleens from 3 animals per group (TLR7.tg6, TLR7.tg6.*Bank1^–/–^*, and control C57BL/6J 32-week-old male mice) were individually homogenized, and RBCs were lysed with ACK buffer. Splenocytes from each animal were processed for single cells in a 10× genomics chromium system at the Institute of Parasitology and Biomedicine López Neyra (CSIC), in Granada, Spain. Raw single-cell count data were obtained using cellranger 10× software. All the analyses were carried out in R using mainly Seurat R package (57) and following the pipeline described by Mathew et al. (32). First, raw data were merged in a single Seurat object in R. Cells with a percentage of mitochondrial counts > 25%, percentage of ribosomal counts > 25%, number of unique features or total counts outside the 0.5%–99.5% range of all cells, number of unique features < 200, or Gini or Simpson diversity index < 0.8, were discarded. In addition, mitochondrial genes and genes expressed in fewer than 5 cells were removed. Total counts per cell were normalized and fixed to 1,000, and gene counts were log transformed. Feature values were standardized by mean centering and SD scaling, and then, values per cell were adjusted correcting by cell cycle scoring and mitochondrial counts. Doublets were identified and removed using scDblFinder R package. Finally, data integration across cells was performed using variance-stabilizing transformation. UMAP was used to cluster the cells. Cluster stability was measured using clustree R package (58). Cells were annotated by correlation with specific cell type and cell subtype markers (32). First, B cells, among the major cell types (T cells, monocytes, etc.) were identified followed by the identification of specific subclusters within the B cells (*Cd19*, *Cd79a*, and *Ms4a1*), performing again the entire analysis process (from the data loading into R) and excluding cells not cataloged as B cells. Trajectory analysis of B cell clusters was carried out using Monocle 3 package.

### Statistics.

All figures and plots except those generated from single-cell data show data points from independent mice pooled across multiple experiments. Survival data were tested by Kaplan-Meyer analysis with significance determined by the log-rank (Mantel-Cox) test. For ELISA and flow cytometry analyses, the Mann-Whitney *U* test was performed in unpaired groups of samples. Data are shown as mean ± SEM. Correlation data were tested by paired Pearson correlation. Prior analysis of the outliers was performed in all the sample cohorts by Tukey’s test. *P* < 0.05 was considered significant. All statistical analyses were performed using GraphPad Prism 8 (GraphPad software Inc.) and R. For the single-cell analysis, the proportion test was applied to measure significant differences in the proportion of cells of each group in each cluster. Differential expression analysis between clusters and/or groups was performed using the Mann-Whitney *U* test with the FindMarkers function from the Seurat R package. Bonferroni-corrected *P* values less than 0.05 were deemed significant. Functional analysis of DEGs was performed using Genecodis (59).

### Study approval.

All the experimental procedures were approved by the Animal Experimentation Committee from the University of Granada and the Ministry of Agriculture of Spain (25/05/2017 and 10/05/2022/063).

### Data availability.

All data supporting the values associated with the main and supplemental findings of this study are reported in the Supporting Data Values file. All original files for scRNA-Seq data have been deposited in NCBI’s Gene Expression Omnibus (GEO), under the accession no. GSE271694 (https://www.ncbi.nlm.nih.gov/geo/query/acc.cgi?acc=GSE271694). All materials generated as part of this study will be made available upon request to the corresponding authors.

## Author contributions

MEAR conceived and conceptualized the study. MEAR supervised the project. GGH and MM designed and performed most of the experiments. GGH and MM carried out all the animal facility maintenance, animal sacrifice, and samples collection. GGH performed the ELISA, designed the flow cytometry panels, carried out the flow cytometry experiments, performed the in vitro cultures, and analyzed the data. GGH and MM performed part of the immunofluorescence experiments. MM supervised the analysis and helped with data interpretation. TD conducted the scRNA-Seq and bioinformatics analysis. GG assisted with the histological, immunofluorescence, and flow cytometry experiments. GGH and MEAR wrote the manuscript. MEAR, MM, GG, and TD provided intellectual contributions throughout the project. MEAR, MM, GG, and TD critically reviewed and edited the manuscript. MEAR provided funding for the study.

## Supplementary Material

Supplemental data

Supplemental data set 1

Supporting data values

## Version 1 08/20/2024

In-Press Preview

## Version 2 10/08/2024

Electronic publication
