# Supplementary material for: Bank1 modulates the differentiation and molecular profile of key B cell populations in autoimmunity
Source: JCI Insight. 2024 Aug 20;9(19):e179417. doi: 10.1172/jci.insight.179417 (PMC11466193; doi:10.1172/jci.insight.179417)
Supplement: Supplemental data [file jciinsight-9-179417-s121.pdf]

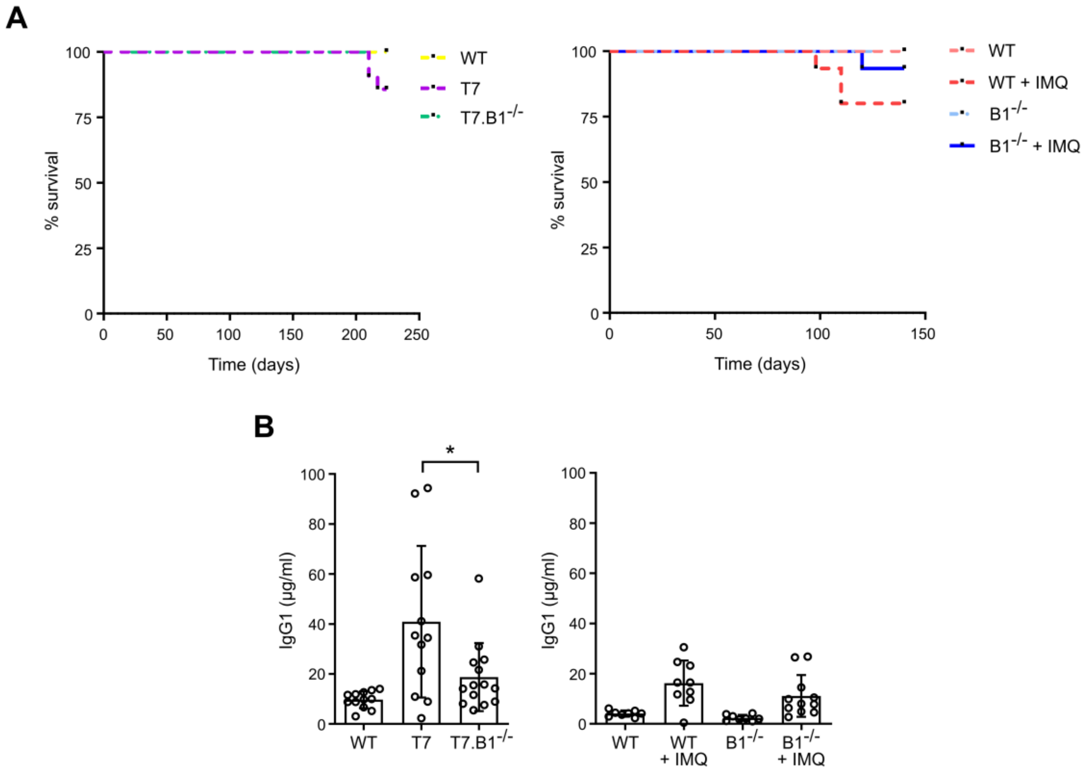


**Fig. S1. *Bank1* deficiency barely modifies the survival rate in the TLR7.Tg6 and in IMQ-treated mice.** (**A**) Survival curve was monitored in both models. TLR7.Tg6 model with WT (n = 12), T7 (n = 21) and T7.B1$^{-/-}$ (n = 15) mice up to 32 weeks; and IMQ-induced model with WT (n = 12), WT + IMQ (n = 15), B1$^{-/-}$ (n = 12) and B1$^{-/-}$ + IMQ (n = 15) mice up to 20 weeks. A Kaplan-Meier survival plot is shown. (**B**) Serum levels of IgG1 (µg/ml) at 1:10000 dilutions. Total mice analyzed: WT (n = 12), T7 (n = 12), T7.B1$^{-/-}$ (n = 14); and WT (n = 8), WT + IMQ (n = 9), B1$^{-/-}$ (n = 8), B1$^{-/-}$ + IMQ (n = 11). Each point represents one individual mouse. Data are shown as mean ± SEM. Mann-Whitney U test with Welch's correction was used to test statistical significance.

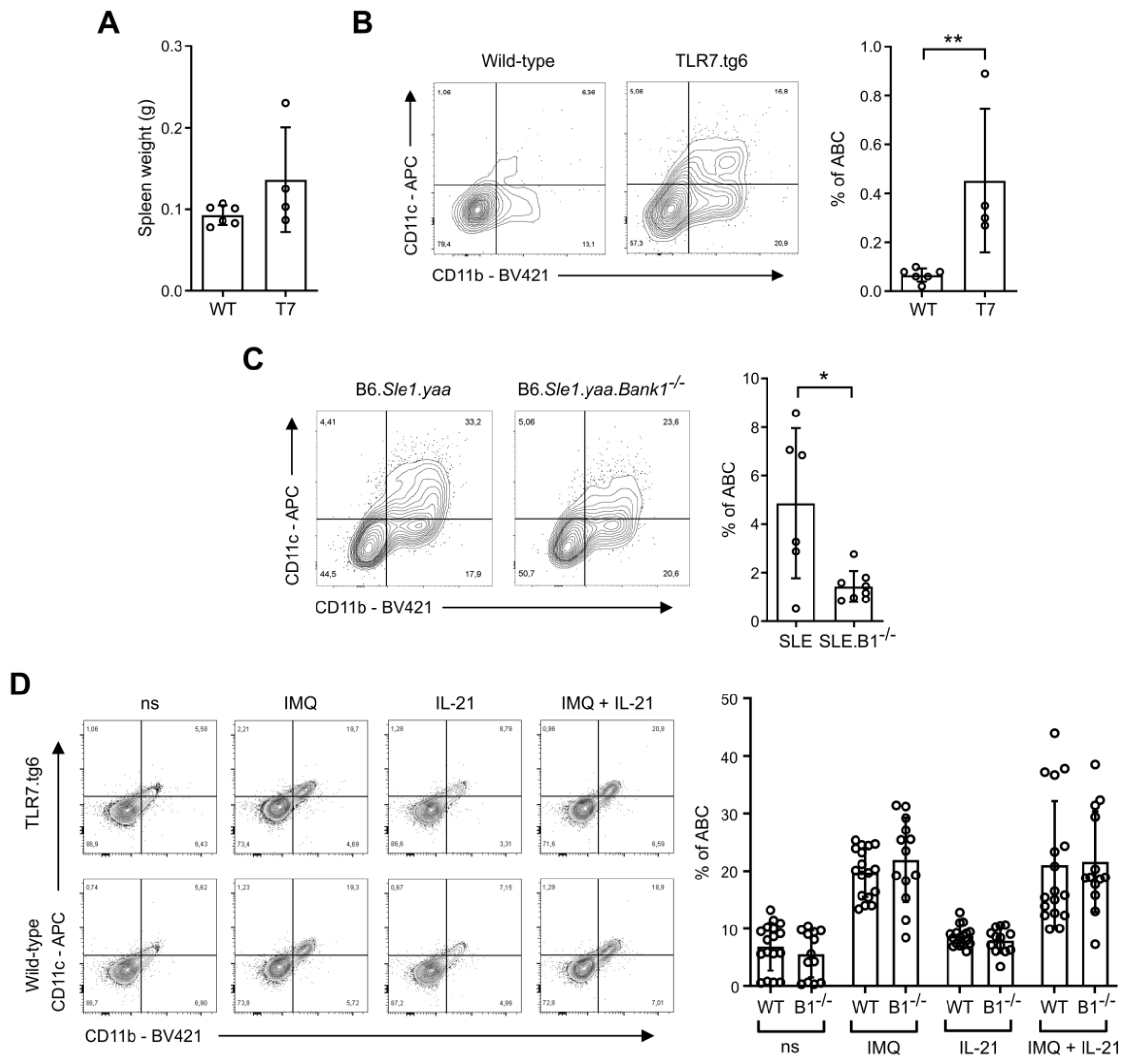


**Fig. S2. Frequency of ABCs at early ages, in the B6.*Sle1.yaa* model and *in vitro* differentiation.** (**A**) Spleen weight from wild-type (n = 6) and TLR7.tg6 (n = 4) mice at 10 weeks of age. (**B**) Frequency of ABCs among CD19$^+$ B cells from the spleens of wild-type and TLR7.tg6 mice at 10 weeks of age. Total mice analyzed: WT (n = 6), T7 (n = 4). (**C**) Frequency of ABCs among CD19$^+$ B cells from the spleens of B6.*Sle1*.*yaa* and B6.*Sle1*.*yaa*.*Bank1*$^{-/-}$ mice at 36 weeks of age. Total mice analyzed: SLE (n = 6), SLE.B1$^{-/-}$ (n = 8). (**D**) Frequency of ABCs (CD11b$^+$ CD11c$^+$) among CD19$^+$ T-bet$^+$ B cells from cultures of B cells purified from wild-type and TLR7.tg6 10 week-old mice, stimulated with 5 μg/ml F(ab')2-goat anti-mouse IgM, 5 μg/ml Ultra-LEAF purified anti-

mouse CD40, in presence or absence of 2.5 µg/ml imiquimod, 25 ng/ml mouse IL-21 recombinant, and the combination of both, for 72 hours. Graph shows mean and individual values of 3 independent experiments. Total mice analyzed: WT (n = 17), B1$^{-/-}$ (n = 13). Each point represents one individual mouse. Data are shown as mean ± SEM. Mann-Whitney U test with Welch's correction was used to test statistical significance.

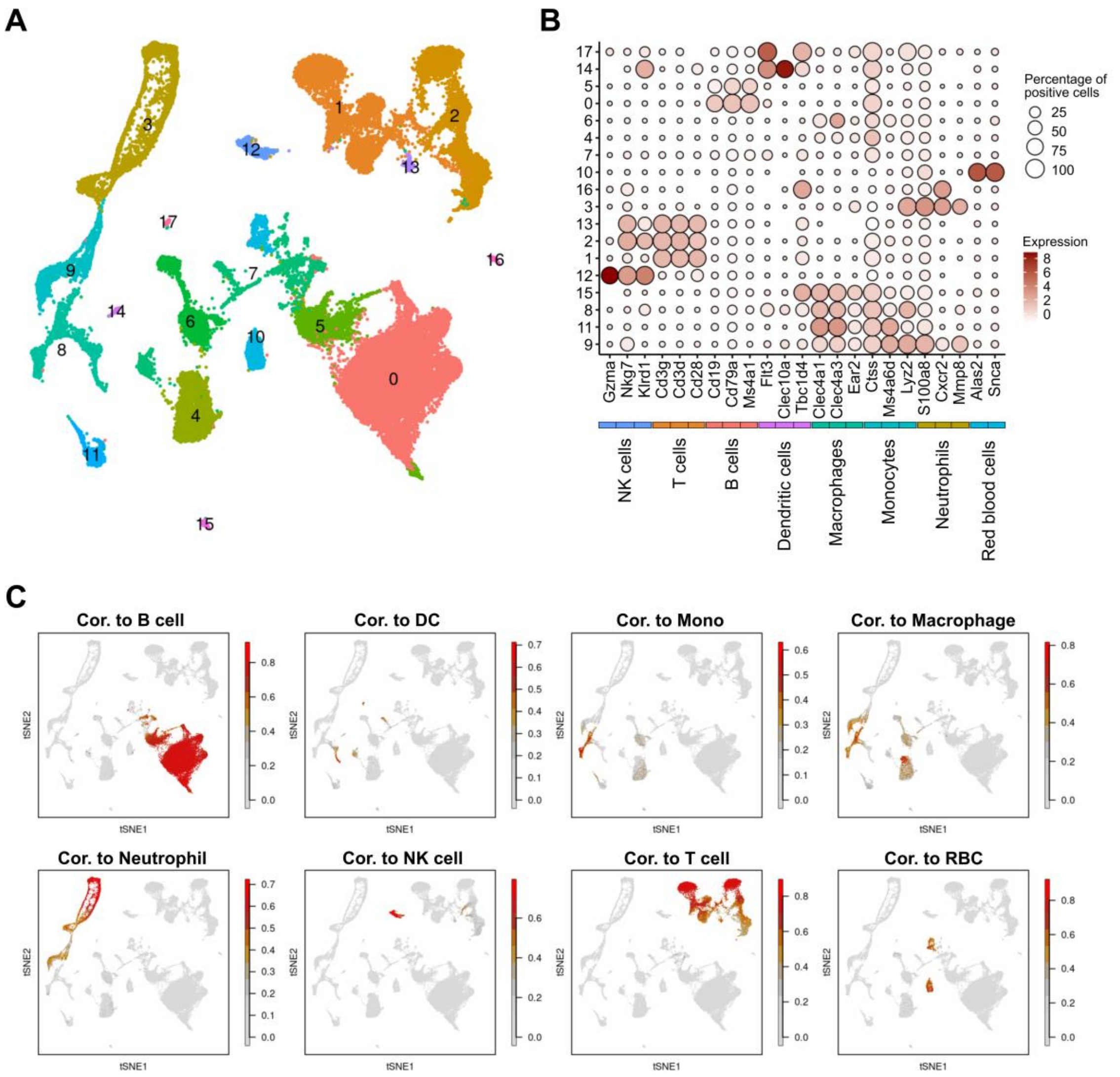


**Fig. S3. Clustering of scRNA-Seq data**. (**A**) UMAP showing clusters of main cell types of spleen. Each point is a cell and the colours represent the different clusters. (**B**) Percentage of cells with the positive expression for cell type-specific markers (circle size) and their mean of expression (colour) across cells from each clusters. The markers are in columns and clusters are in rows. (**C**) Correlation between a panel of cell-specific markers and their expression by cells. Correlation is represented by the colour scale.

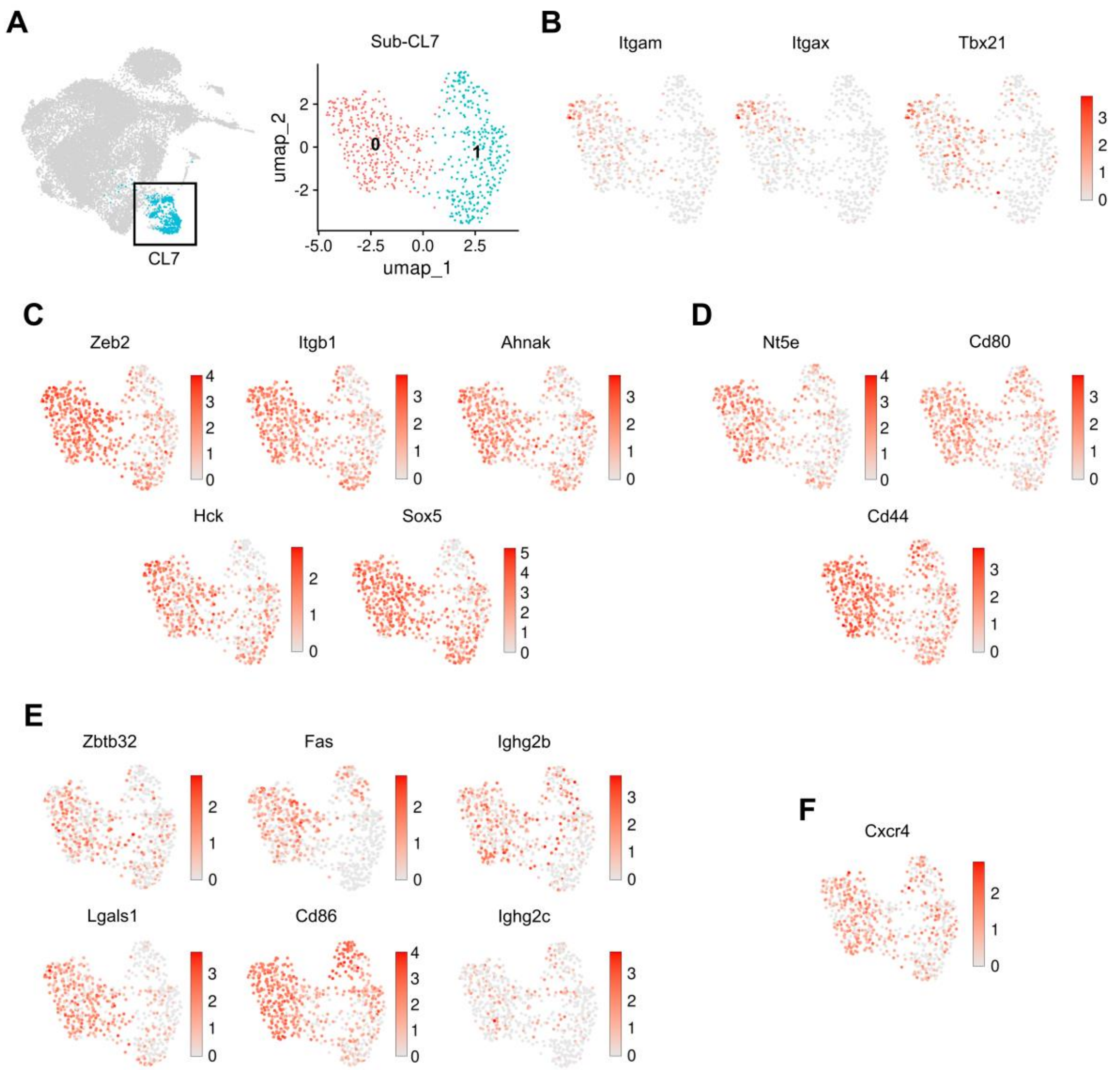


**Fig. S4. Subclusterization of CL7**. (**A**) UMAP showing two different subclusters (subCL7-0 and subCL7-1) obtained from the subclusterization of CL7. (**B**) Individual UMAPs showing the expression levels of typical markers of ABCs. (**C**) Individual UMAPs showing the expression levels of typical markers of: ABCs and atypical memory B cells (atMBCs), (**D**) memory B cells (MBCs), (**E**) plasma cells (PCs) and (**F**) extrafollicular cells.

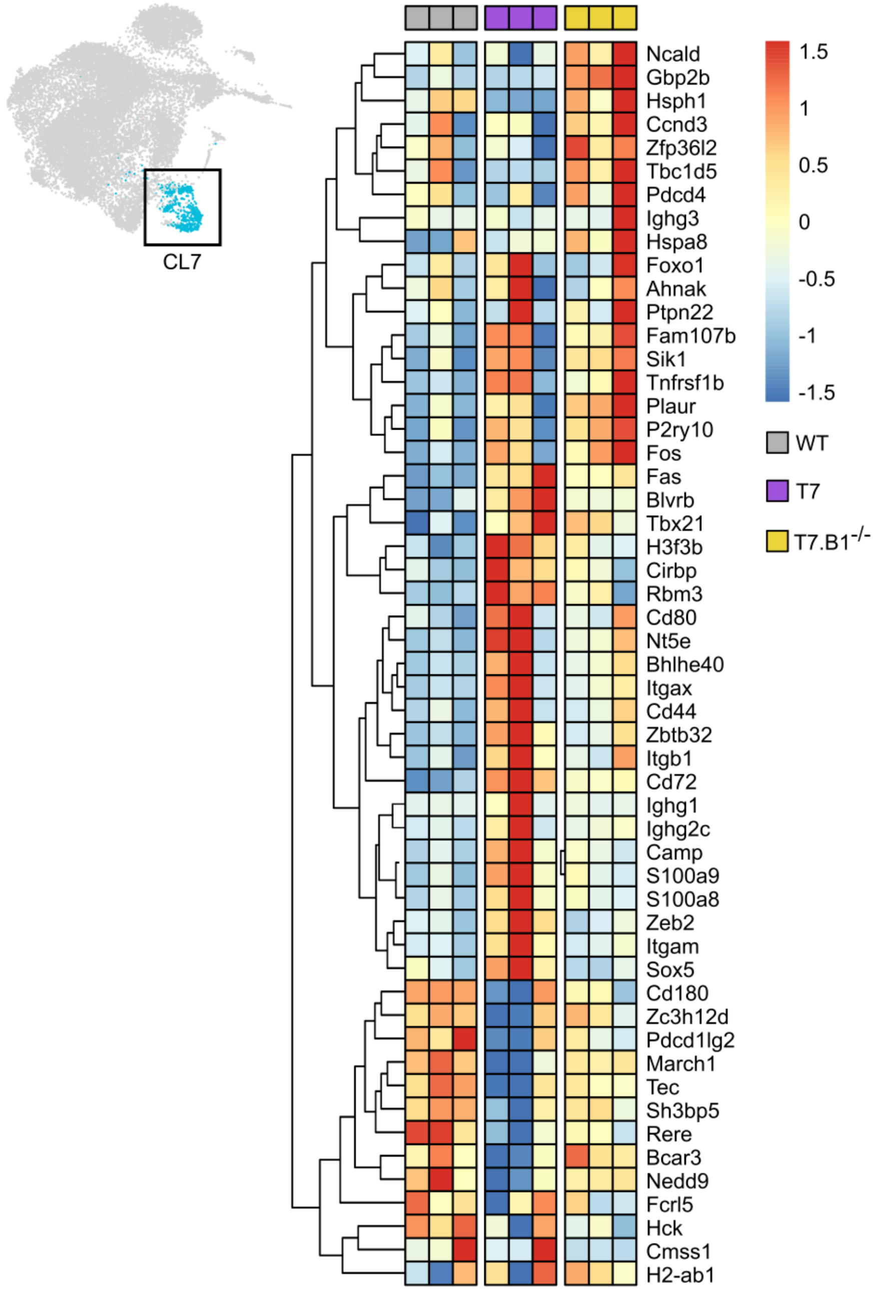


**Fig. S5. *Bank1* deficiency modified the differentially expressed genes in cluster 7.** Heatmap showing the differentially expressed genes between wild-type, TLR7.tg6 and TLR7.tg6.Bank1-/- mice, in cluster 7. Colour represent the gene expression normalized by z-score.

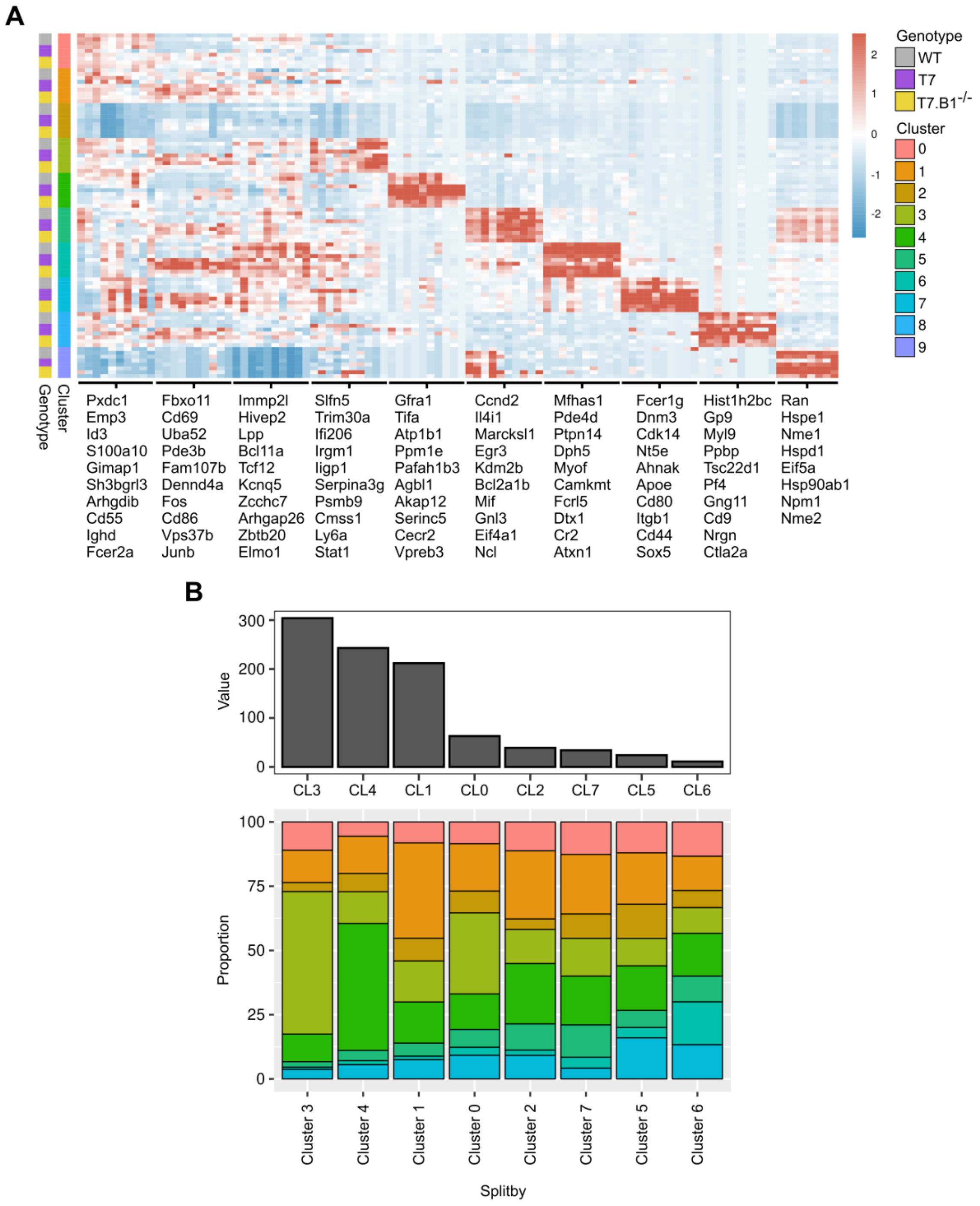


**Fig. S6. scRNA-Seq transcriptome data from 10 clusters among CD19$^+$ B cells**. (**A**) Top 10 most differentially expressed genes comparing each B cell clusters against the rest of B cell clusters. Colour scale represent the gene expression normalized by z-score. (**B**) Above is the number of DEGs comparing each cluster against the rest. The proportion of significant genes unique and shared with other clusters is shown below.

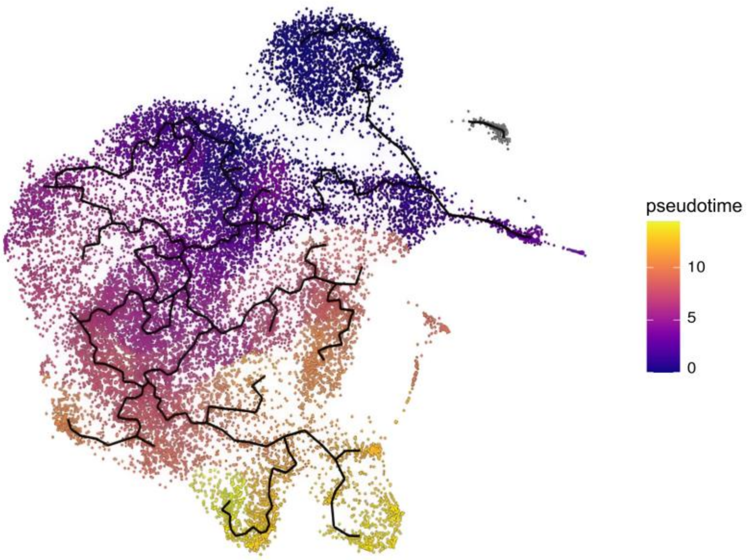


**Fig. S7. Alternative trajectory of splenic B cells from the TLR7.tg6 model**. UMAP visualization of the clusters arranged along trajectories, with CL2 as initial pseudotime (root), coloured by inferred pseudotime, calculated by Monocle3.

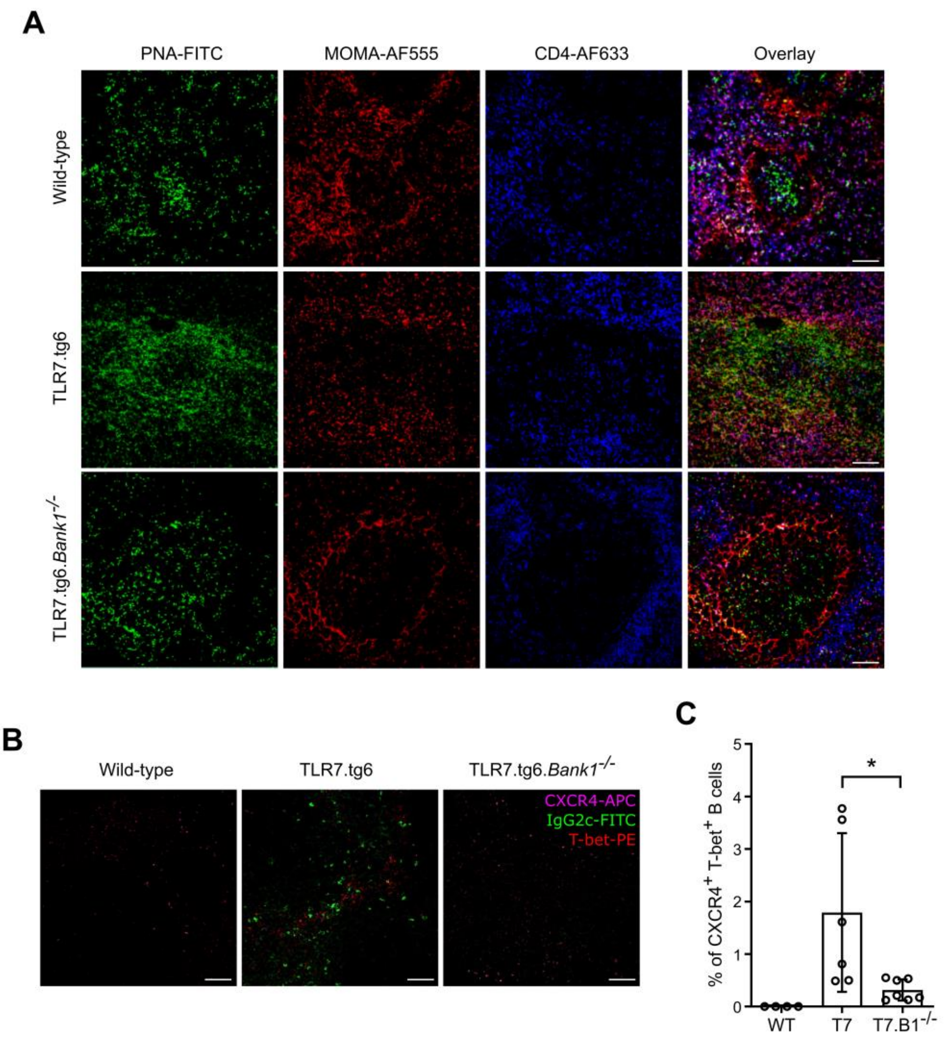


**Fig. S8. T-bet$^+$ extrafollicular B cells are increased in TLR7.tg6 mice compared to *Bank1*-deficient and wild-type mice**. (**A**) Representative cryosections of spleens from 32 week-old wild-type, TLR7.tg6 and TLR7.tg6.*Bank1*$^{-/-}$ mice, stained with anti-PNA FITC, anti-MOMA-1 conjugated with Alexa Fluor 555, and anti-CD4 conjugated with Alexa Fluor 633. Scale bar: 100 µm. This representative experiment was conducted 3 different times. (**B**) Representative cryosections of spleens from 32 week-old wild-type, TLR7.tg6 and TLR7.tg6.*Bank1*$^{-/-}$ mice, stained with anti-CXCR4 APC, anti-IgG2c FITC and anti-T-bet PE. Scale bar: 100 µm. This representative experiment was conducted 3 different times. (**C**) Frequency of CXCR4$^+$ T-bet$^+$ cells among IgG2c$^+$ CD138$^+$ B220$^-$

cells from the spleens of 32 week-old TLR7.tg6 model. Total mice analyzed: WT (n = 4), T7 (n = 6), T7.B1$^{-/-}$ (n = 7). Each point represents one individual mouse. Data are shown as mean ± SEM. Mann-Whitney U test with Welch's correction was used to test statistical significance.

## Legends for data files (Excel files):

**Data file S1. Significantly expressed genes comparing between clusters**. ClusterReference: cluster for which the rest of the values of the same row refer; GeneID: Gene symbol identifier; PvalAdj: p-value adjusted by Bonferroni; AvgLog2FC: fold change in base 2 logarithmic scale; pct1: percentage of cells expressing the gene in the studied cluster; pct2: percentage of cells expressing the gene in the reference cluster/clusters.

**Data file S2. Significantly expressed genes comparing between subclusters from CL7.** ClusterReference: cluster for which the rest of the values of the same row refer; GeneID: Gene symbol identifier; PvalAdj: p-value adjusted by Bonferroni; AvgLog2FC: fold change in base 2 logarithmic scale; pct1: percentage of cells expressing the gene in the studied cluster; pct2: percentage of cells expressing the gene in the reference cluster/clusters.

**Data file S3. Significantly expressed genes comparing TLR7.tg6 and TLR7.tg6.*Bank1*$^{-/-}$ by cluster**. Each excel sheet contains the results obtained for a cluster. GeneID: Gene symbol identifier; PvalAdj: p-value adjusted by Bonferroni; AvgLog2FC: fold change in base 2 logarithmic scale; Comparation: group of mice compared. Second group as used as reference for the statistical test.

**Data file S4. Significant biological pathways comparing between clusters**. ClusterReference: cluster for which the rest of the values of the same row refer; PvalAdj: p-value adjusted by Bonferroni; Direction: significant biological pathway for up-

regulated or downregulated genes; Description: pathway description; AnnotationID: Gene Ontology identifier.

**Data file S5. Significant biological pathways comparing TLR7.tg6 and TLR7.tg6.*Bank1*$^{-/-}$ by cluster**. ClusterReference: cluster for which the rest of the values of the same row refer; Direction: significant biological pathway for up-regulated or downregulated genes; PvalAdj: p-value adjusted by Bonferroni; Description: pathway description; AnnotationID: Gene Ontology identifier.
